# The Q Exactive HF, a Benchtop Mass Spectrometer with a Pre-filter, High-performance Quadrupole and an Ultra-high-field Orbitrap Analyzer* [Fn FN2]

**DOI:** 10.1074/mcp.M114.043489

**Published:** 2014-10-30

**Authors:** Richard Alexander Scheltema, Jan-Peter Hauschild, Oliver Lange, Daniel Hornburg, Eduard Denisov, Eugen Damoc, Andreas Kuehn, Alexander Makarov, Matthias Mann

**Affiliations:** From the ‡Department of Proteomics and Signal Transduction, Max Planck Institute of Biochemistry, Am Klopferspitz 18, D-82152 Martinsried, Germany;; §Thermo Fisher Scientific (Bremen) GmbH, Hanna-Kunath-Strasse 11, 28199 Bremen, Germany

## Abstract

The quadrupole Orbitrap mass spectrometer (Q Exactive) made a powerful proteomics instrument available in a benchtop format. It significantly boosted the number of proteins analyzable per hour and has now evolved into a proteomics analysis workhorse for many laboratories. Here we describe the Q Exactive Plus and Q Exactive HF mass spectrometers, which feature several innovations in comparison to the original Q Exactive instrument. A low-resolution pre-filter has been implemented within the injection flatapole, preventing unwanted ions from entering deep into the system, and thereby increasing its robustness. A new segmented quadrupole, with higher fidelity of isolation efficiency over a wide range of isolation windows, provides an almost 2-fold improvement of transmission at narrow isolation widths. Additionally, the Q Exactive HF has a compact Orbitrap analyzer, leading to higher field strength and almost doubling the resolution at the same transient times. With its very fast isolation and fragmentation capabilities, the instrument achieves overall cycle times of 1 s for a top 15 to 20 higher energy collisional dissociation method. We demonstrate the identification of 5000 proteins in standard 90-min gradients of tryptic digests of mammalian cell lysate, an increase of over 40% for detected peptides and over 20% for detected proteins. Additionally, we tested the instrument on peptide phosphorylation enriched samples, for which an improvement of up to 60% class I sites was observed.

Mass spectrometry (MS)-based^1^ proteomics aims at the comprehensive analysis of proteins present in a biological sample (1), and the field has expanded in many surprising directions (2). Application of the developed techniques has revealed novel insights into fundamental biology, as well as produced analysis techniques with implications for clinical applications. A major hurdle, however, is the complexity of the systems under scrutiny, as it has been shown that human cell lines, for instance, express at least 10,000 genes that are detectable as proteins (3–5). If we further consider all the peptides produced in bottom-up proteomics experiments, this hurdle is compounded, as ideally many hundreds of thousands of analytes should be characterized in order for the proteins giving rise to them to be fully reconstructed (6). In principle, issues of sample complexity and dynamic range could be addressed by a very high degree of up-front fractionation. However, this strategy faces diminishing returns and leads to unacceptably long analysis times for most purposes. Given the fact that even with optimal chromatographic resolution many peptides with abundance differences of many orders of magnitude elute within the same time frame, there remains a need to improve the mass spectrometric detection in terms of speed, resolution, and sensitivity.

Nanoscale liquid chromatography coupled online to mass spectrometry is the current technique of choice for the analysis of complex peptide mixtures. In a top-*N* shotgun strategy, a full scan, providing a complete overview of isotope patterns resulting from ionized peptides, is followed by *N* fragmentation scans performed on the most abundant not-yet-sequenced isotope patterns currently visible in the full scan. During fragmentation, the goal is to cleanly isolate the intended precursor peptide ion, which today is generally done either by a linear ion trap or by a quadrupole mass filter. Fragment ions are then mass measured by an Orbitrap mass analyzer, a time-of-flight analyzer, or, less often, ion cyclotron resonance–Fourier transform or linear or three-dimensional ion traps.

Apart from the MS instrumentation, recent developments in the proteomics workflow include a move toward automated online quality control systems (7, 8) and single-run analyses (9), which require very high-performance peptide chromatography (10, 11).

The Orbitrap mass analyzer was introduced commercially almost 10 years ago, and hybrid instruments based on this tool have become very popular in proteomics (12). They consist of an upfront mass spectrometer coupled to a so-called C-trap, which stores and compresses the ion population (generally up to one million charges) prior to injection into the Orbitrap analyzer. Up to the Orbitrap Velos and Elite members of this family of instruments, the precursor selection (and usually the fragmentation) occurred in the linear ion trap (13), but a few years ago an instrument based on a quadrupole front end—the Q Exactive mass spectrometer—was developed (14). Compared with the linear ion trap, quadrupole mass filters have the advantage of being capable of nearly instantaneously selecting a small mass region by modulating the RF field, allowing only a select set of ions to have stable trajectories when passing through the rod assembly. As we described previously, this near-instantaneous mass selection capability and the C-trap's capability of storing ions enable multiplexing of different ion populations (*e.g.* fragment ions of two or more distinct precursor ions) prior to analysis in the Orbitrap mass analyzer (14). However, that instrument did not use the highest efficiency quadrupole technology (15), and it did not use the compact high-field Orbitrap analyzer that had been introduced in the Orbitrap Elite instrument (16).

Here we describe the advances incorporated into the Q Exactive Plus and the Q Exactive HF instruments. These include improved robustness effected by a low-resolution filter upstream of the quadrupole, a segmented quadrupole, and, in the case of the Q Exactive HF instrument, an ultra-high-field mass Orbitrap analyzer, doubling resolution or acquisition speed. We describe these capabilities in the context of single-shot complex mixture analysis of peptides and phosphopeptides.

## EXPERIMENTAL PROCEDURES

### 

#### 

##### Construction of the Q Exactive Plus

**Fig. 1.**
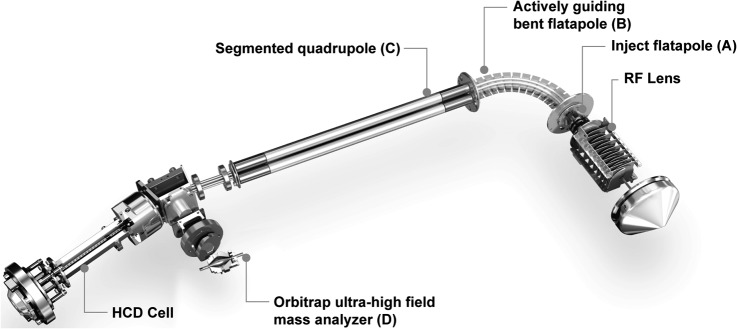
**Construction details of the Q Exactive HF.** This instrument is based on the Q Exactive series and improves on it with a mass selection pre-filter implemented at the injection flatapole (*A*), an actively guiding bent flatapole (*B*), and a segmented quadrupole (*C*). This combination prevents contamination from traveling far into the instrument and improves the ion transmission almost 2-fold. The ultra-high-field Orbitrap analyzer (*D*) is optional.

The instrument is based on the previous-generation Q Exactive mass spectrometer (14) with the same general design elements. Briefly, these consist of an atmospheric pressure ion source, a stacked-ring ion guide (S-lens) and an injection flatapole in the source region, a bent flatapole containing a large bore at the bend ejecting solvent droplets and other neutral species to prevent them from entering further into the instrument, a segmented quadrupole mass filter, a C-trap, a higher energy collisional dissociation cell, and an Orbitrap mass analyzer (Fig. 1). In contrast to the previous generation, the injection flatapole was equipped with ion selection capabilities, providing a low-resolution selection mechanism for the removal of undesirable ions from the ion beam before higher resolution selection in the quadrupole mass filter. The new, segmented quadrupole mass filter has improved ion transmission and mass selection characteristics (see below). Lastly, the instrument can optionally be equipped with a compact Orbitrap analyzer (Q Exactive HF), leading to a higher field and consequently higher frequency of ion motion. This in turn leads to almost twice the resolution in the same scan time relative to the previous cell (16). Alternatively, this analyzer can be used to achieve twice the scan speed while remaining at nearly the same resolution, a strategy we utilize here.

The covered mass range in the Orbitrap analyzer is *m*/*z* 50–6000. Precursor mass selection by the quadrupole is possible up to *m*/*z* 2500, and isolation windows can be set between 0.4 and 5600 Th. The instrument software automatically adjusts the required ion injection time to compensate for loss of transmission when reducing the isolation width. Acquisition speed with the classic Orbitrap analyzer remains the same as previously reported, whereas for the high-field Orbitrap it ranges from 27 Hz for resolving power 15,000 specified at *m*/*z* 200 (corresponding to 10,000 at *m*/*z* 400) to 1.5 Hz for resolving power 240,000 at *m*/*z* 200 (corresponding to 170,000 at *m*/*z* 400). The vacuum in the Orbitrap compartment can be electronically adjusted ∼5-fold, enabling high-resolution analysis of most analytes including large peptides and small proteins.

##### Preparation of HeLa Lysates

HeLa cells (ATCC, S3 subclone) were cultured in DMEM containing 10% fetal bovine serum, 20 mm glutamine, and 1% penicillin-streptomycin. Cells were collected via centrifugation at 200*g* for 10 min, washed once with cold PBS, and centrifuged again. Supernatant was carefully discarded, and cell pellets were shock frozen in liquid nitrogen and stored at −80 °C. Aliquots of ∼3e7 cells were re-suspended in 1 ml of water; then 1 ul of trifluoroethanol was added, and samples underwent incubation for 10 min on ice, a 2-min sonication at duty cycle 30% and output control 3 (Branson, Danbury; sonifier model 250), and 1 min of vortexing. After 20 min of incubation at 56 °C, 25 μl of 200 mm DTT was added to reduce proteins, and samples were incubated at 90 °C for 15 min. Alkylation was then performed by adding 100 μl of 200 mm iodoacetamide and incubating for 60 min at room temperature in the dark. The sample was diluted using 8 ml of 50 mm NH_4_HCO_3_ to reduce the final trifluoroethanol concentration to 10% (v/v), after which the sample was digested for 1 h at 37 °C by LysC at an enzyme:protein ratio of 1:100 and then overnight at 37 °C after the addition of trypsin at a ratio of 1:100. Digests were then diluted 1:4 with 0.1% formic acid (v/v) and purified with Sep-Pak tC_18_ cartridges according to the manufacturer's instructions. The peptide concentration was determined using a NanoDrop spectrophotometer (Thermo Scientific).

##### Phosphorylation Enrichment of Non-stimulated HeLa Cells

Peptides were collected from a population of 1e8 unstimulated HeLa cells prepared using the filter-aided sample preparation method (17). In brief, cell pellets were solubilized in 4% SDS, 100 mm Tris/HCl, pH 7.6, 0.1 m DTT; incubated at 95 °C for 5 min; and sonicated at duty cycle 30% and output control 3 (Branson Ultrasonics). The protein concentration was determined from tryptophan fluorescence emission at 350 nm using an excitation wavelength of 295 nm. A total of 30 mg of protein extract was then split on top of five 30,000 molecular weight cutoff centrifugal filters (20 mg per filter), spun down, and washed twice with 7 ml of 8 m urea, 100 mm Tris/HCl, pH 8.5. Alkylation was performed with 50 mm iodoacetamide for 30 min at room temperature in the dark in the same buffer. After two further washes with 7 ml of 8 m urea, Tris/HCl, pH 8.5, in 0.1 m and three with 7 ml of NH_4_HCO_3_, digestion was performed by adding LysC at an enzyme:protein ratio of 1:50 and incubating overnight at 30 °C. The digested peptides were eluted from the filters via centrifugation, quantified with a NanoDrop spectrophotometer, and then further digested by trypsin added at a ratio of 1:100. After incubation at 37 °C for 5 h, peptides were shock frozen in liquid nitrogen and lyophilized. Peptides (around 10 mg per Falcon tube) were re-suspended in 10 ml of ACN 80%, TFA 6%, and insoluble peptides were spun down by centrifugation at 100*g* for 1 min. Supernatants were moved into new 15-ml Falcon tubes, and samples were incubated twice with 50 mg of TiO_2_ beads on a rotating wheel for 45 min. TiO_2_ beads from all the enrichments were then pooled together and washed three times with 12 ml of ACN 80%, TFA 6% and three times with 12 ml of ACN 80%, TFA 0.1%. Beads were then re-suspended in 2 ml of ACN 80%, TFA 0.1%, transferred into 12 Empore-C8 StageTips (18), and washed once with ACN 80%, TFA 0.1%. Peptides were eluted from each StageTip with 200 μl of 60% NH_4_OH (25% NH_3_ solution in H_2_O) in 40% ACN. The volume was reduced via SpeedVac to 10 μl to eliminate ACN and brought back up to 200 μl with 0.1% formic acid. Phosphorylation enriched peptides were pooled and purified with Sep-Pak tC_18_ cartridges according to the manufacturer's instructions. The peptide concentration was determined using a NanoDrop spectrophotometer. The final concentration was brought to 400 ng/μl with 0.1% formic acid, and 5.5-μl aliquots were frozen at −20 °C.

##### LC-MS/MS Analysis

Online chromatography was performed with the Thermo Easy nLC ultra-high-pressure HPLC system (Thermo Fisher Scientific) coupled online to either an original Q Exactive or a Q Exactive HF with a NanoFlex source (Thermo Fisher Scientific). Analytical columns (50 cm long, 75-μm inner diameter) were packed in-house with ReproSil-Pur C_18_ AQ 1.9-μm reversed phase resin (Dr. Maisch GmbH, Ammerbuch-Entringen, Germany) in buffer A (0.5% acetic acid). During on-line analysis the analytical column was placed in a column heater (Sonation GmbH, Biberach, Germany) regulated to a temperature of 55 °C. A peptide mixture of 2 μg dry weight was loaded onto the analytical column with buffer A at a maximum back-pressure of 980 bar (generally resulting in a flow rate of 450 nL/min) and separated with a linear gradient of 5% to 30% buffer B (80% ACN and 0.5% acetic acid) at a flow rate of 250 nL/min controlled by IntelliFlow technology over 90 min (generally at a back-pressure of around 500 bar). Due to the loading, lead-in, and washing steps, the total time for an LC-MS/MS run was about 40 to 50 min longer. Online quality control, including automated detection of large droplet formation, HPLC parameters, and acquisition-related computer status, was performed with SprayQc (8).

MS data were acquired using a data-dependent top-10 method for the Q Exactive and a top-15 method for the Q Exactive HF, dynamically choosing the most abundant not-yet-sequenced precursor ions from the survey scans (300–1650 Th). Sequencing was performed via higher energy collisional dissociation fragmentation with a target value of 1e5 ions determined with predictive automatic gain control (supplemental Fig. S6). Isolation of precursors was performed with a window of 3 Th for the Q Exactive and 1.4 Th for the Q Exactive HF, because of the latter's superior quadrupole (Fig. 3*C*). Survey scans were acquired at a resolution of 70,000 at *m*/*z* 200 on the Q Exactive and 60,000 at *m*/*z* 200 on the Q Exactive HF (“Results and Discussion”). Resolution for HCD spectra was set to 17,500 at *m*/*z* 200 with a maximum ion injection time of 120 ms on the Q Exactive and 15,000 at *m*/*z* 200 with maximum ion injection time of 25 ms on the Q Exactive HF (supplemental Fig. S3). The normalized collision energy was 25 for the Q Exactive and 27 for the Q Exactive HF (supplemental Fig. S2; this difference was due to different scaling functions in the instrument software). The “underfill ratio,” specifying the minimum percentage of the target ion value likely to be reached at the maximum fill time, was defined as 10% (supplemental Fig. S5). Furthermore, the S-lens RF level was set at 60, which gave optimal transmission of the *m*/*z* region occupied by the peptides from our digest (supplemental Fig. S4). We excluded precursor ions with single, unassigned, or six and higher charge states from fragmentation selection (supplemental Fig. S2).

In the comparison between the Q Exactive and the Q Exactive HF, we noticed that there was a 1.5-times overestimation of the number of ions offered for fragmentation on the Q Exactive HF relative to the Q Exactive runs. To determine whether this had an additional effect on the performance of the instrument, we investigated performance on different target values. From this we found that the original Q Exactive target value of 1e5 ions remained optimal for the Q Exactive Plus and Q Exactive HF (supplemental Fig. S6).

##### Data Analysis

All data were analyzed with the MaxQuant proteomics data analysis workflow, version 1.4.0.6 (19), with the Andromeda search engine (20). The false discovery rate was set at 1% for protein, peptide spectrum match, and site decoy fraction levels. Peptides were required to have a minimum length of seven amino acids and a maximum mass of 4600 Da. MaxQuant was used to score fragmentation scans for identification based on a search with an allowed mass deviation of the precursor ion of up to 4.5 ppm after time-dependent mass calibration. The allowed fragment mass deviation was 20 ppm. Fragmentation spectra were searched by Andromeda in the International Protein Index human database (version 3.68; 87,061 entries) combined with 262 common contaminants (20). Enzyme specificity was set as C-terminal to arginine and lysine, also allowing cleavage at proline bonds and a maximum of two missed cleavages. We set carbamidomethylation of cysteine as a fixed modification and N-terminal protein acetylation and oxidation (M) as variable modifications for the HeLa total cell lysates; additionally, Phospho (STY) was set as a variable modification for the phosphorylation enriched samples. Further downstream analysis of the results was performed with in-house-developed tools for the extraction of metadata from the mass spectrometry files based on MSFileReader (Thermo Fisher Scientific) and with the R scripting and statistical environment (21) using ggplot (22) for data visualization. The datasets used for analysis have been deposited at the ProteomeXchange Consortium via the PRIDE partner repository.

## RESULTS AND DISCUSSION

The ion path during quadrupole isolation of the Q Exactive HF (as well as the Plus) has been updated with the objective of improving robustness and optimizing the ion transmission during quadrupole isolation in order to be able to meet the demand created by the increased speed of the ultra-high-field Orbitrap mass analyzer. Various hardware components making up the path are detailed in “Experimental Procedures.” We start our discussion by investigating their behavior using the standard ESI Positive Ion Calibration Solution (Thermo Fisher Scientific) electrosprayed by direct infusion. We then characterize the performance of the instrument based on results from HeLa whole cell lysates measured via shotgun top-*N* methods.

### 

#### 

##### Injection Flatapole as Pre-filter

**Fig. 2.**
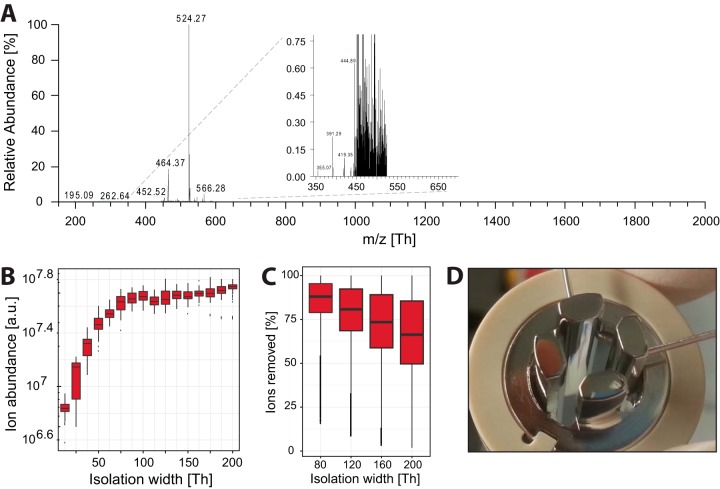
**Performance details of the injection flatapole pre-mass filter.** *A*, the calmix spectrum when isolating MRFA (524.27 *m*/*z*) in an isolation window of 80 Th. The inset zooms in on the isolation window and its direct surroundings. *B*, isolation efficiency of the pre-mass filter over a range of isolation windows. *C*, effectiveness of the pre-mass filter over a range of isolation windows on a HeLa whole cell lysate. *D*, picture of the injection flatapole after 3 months of continuous measurements.

We and others had found that due to the intense peptide ion beam, the Q Exactive could be prone to contamination issues under heavy load that degraded its performance after a prolonged period of measurements. The leading cause was determined to be peptides excluded during mass selection coating the rods of the quadrupole. Therefore, the first update was made to the injection flatapole with the objective of making the instrument more robust. This component now acts as a low-resolution quadrupole capable of rough pre-filtering of the incoming ion beam before proper precursor selection in the analytical quadrupole, thereby providing an protective filter for contaminants. We tested the pre-filter on the tetra-peptide MRFA present in the electrospray ionization calmix solution (Fig. 2*A*). When the pre-filter was activated for the MRFA peak at 524.27 Th in an isolation window of 80 Th, a clean exclusion of peaks was achieved outside the selected isolation range. This is evident from the inset zoom-in to below 1% on the relative ion abundance scale. To determine the isolation width at which the pre-filter has optimal ion transmission, we started with a 10-Th selection window and increased it in 10-Th steps to 200 Th. The signal increased with increasing window size until 80 Th, after which hardly any increase in transmitted ions occurred (Fig. 2*B*). At this isolation width more than 90% of the used *m*/*z* scan-range (300–1650 Th) was excluded. By comparing the summed ion abundance of the signals from a HeLa total cell digest that were discarded when employing this inject flatapole setting to the total ion current, we estimated that over 75% of the ion beam was excluded, for more than 75,000 of over 90,000 fragmentation scans (Fig. 2*C*). (Note that these numbers are somewhat dependent on the *m*/*z* range chosen.) With this low-resolution pre-filtering, a large portion of the selection load is moved to the robust inject flatapole. This, in turn, makes the task for the analytical quadrupole easier and should lead to less deposition on the selection quadrupole. The majority of non-precursor peptide ions are instead deposited on the injection flatapole (Fig. 2*D*), which is located in the outer cage and is readily accessible for cleaning.

##### Segmented Quadrupole

**Fig. 3.**
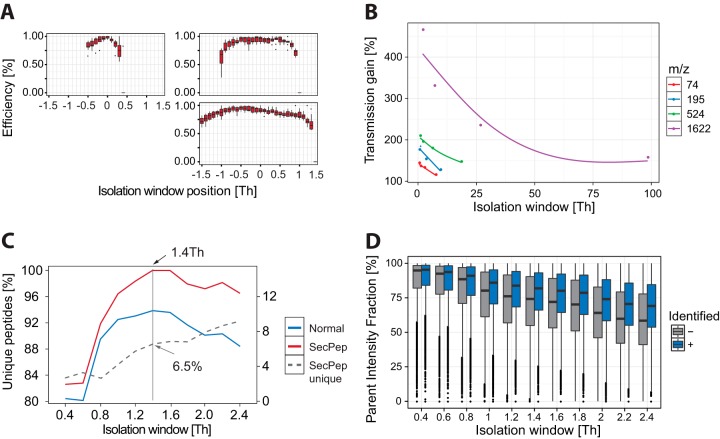
**Transmission characteristics mass selection filter.** *A*, isolation window efficiency comparison between the Q Exactive and Q Exactive Plus using the complete set of calmix ions. *B*, isolation transmission efficiency comparison between the Q Exactive and the Q Exactive HF using the complete set of calmix ions. *C*, total unique peptides from a complex HeLa total cell lysate, sequenced with a range of isolation windows. The secondary *y*-axis denotes the contribution of the Andromeda option “second peptide” as a percentage (SecPep). *D*, isolation purity from a complex HeLa total cell lysate, sequenced with a range of isolation windows.

The original hyperbolic-rod quadrupole has been replaced by a segmented version capable of achieving more rectangular isolation efficiency over the complete isolation window (Fig. 3*A*). This is of relevance for acquisition strategies like SWATH (23) and co-isolation of SILAC partners (for example, in selected ion monitoring scans), which require rectangular isolation windows in order to generate accurate quantitative information at their edges. Relative to the quadrupole in the Q Exactive, we observed markedly improved isolation efficiency at the low-mass side of the isolation window. Additionally, the transmission of the quadrupole for narrow isolation windows has been improved. We measured this improved efficiency for the set of calmix ions and found an almost 2-fold increase (Fig. 2*B*). Note that the figure also shows a transmission benefit for larger isolation windows, especially for high-*m*/*z* ions, that is due to the focusing effect of the exit segment of the quadrupole. This improvement roughly corresponds to the increase in ion current needed to support the doubling of the scan speed that the Orbitrap high-field analyzer is capable of (because the fill time corresponds to the transient time in fully parallel operation of the instrument).

Previously, the smallest isolation windows that we used were about 2.2 Th because transmission declined drastically below this value (24). As the segmented quadrupole also promised the ability to narrow isolation windows, we tested a range of window sizes on a complex HeLa whole cell lysate. We analyzed the data both with and without the “second peptide” option in Andromeda, which attempts to identify a second peptide from already identified MS2 scans after removal of the fragments associated with the first peptide (20). The Q Exactive HF performed optimally in terms of peptide identifications at an isolation window of 1.4 Th both with and without the second peptide option (Fig. 3*C*), whereas for the Q Exactive this value was 2.2 Th. The narrower isolation window also resulted in a reduced addition to the unique peptide sequences given by the second peptide option, because of the lower likelihood of co-isolated peptides that can be identified in this second step. At the optimal window of 1.4 Th this was 6.5%, down from 8% at the former optimal isolation window of 2.2 Th. We determined the purity of isolation by calculating the precursor ion fraction (24) for the same range of isolation windows. At the narrowest isolation window of 0.4 Th, the majority of isolations achieved greater than 80% purity (Fig. 3*D*). Such a low precursor ion fraction might to some degree be helpful for tandem mass tag labeling experiments (25, 26). For the optimal isolation window of 1.4 Th, the precursor ion fraction was slightly reduced to 70% in the large majority of cases. As this is still well within the range of tolerance for a clean and identifiable fragmentation spectrum, we adopted this isolation value as the default.

##### Performance of the Ultra-high-field Analyzer

**Fig. 4.**
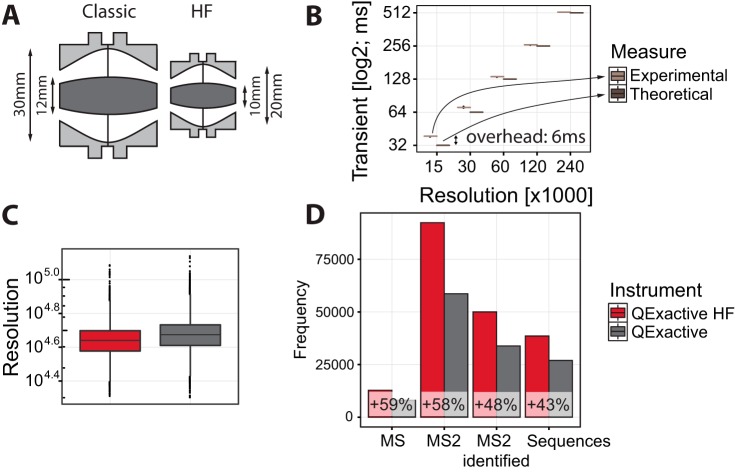
**Performance of the Q Exactive HF with ultra-high-field Orbitrap analyzer.** *A*, comparison of dimensions of the standard (*left*) to the compact, ultra-high-field Orbitrap analyzer (*right*). *B*, comparison of the required Orbitrap transient times required for the different resolutions, together with an estimation of the overhead time required for each scan. *C*, measured resolution of peaks detected from a complex HeLa total cell lysate. *D*, scan statistics comparison between the Q Exactive and the Q Exactive Plus on HeLa total cell lysates.

The ultra-high-field Orbitrap analyzer consists of an outer barrel-like electrode (dark gray in Fig. 4*A*) of maximum radius R2 and a central spindle-like electrode (light gray) along the axis of maximum radius R1, with the outer electrode maintained at the virtual ground of the preamplifier and the central electrode at a voltage −Ur (Ur > 0 for positive ions) (16). In a standard Orbitrap analyzer, R1 = 6 mm and R2 = 15 mm (5), whereas the ultra-high-field analyzer is more compact, with R1 = 5 mm and R2 = 10 mm (Fig. 4*A*) (*i.e.* the outer electrode is scaled down by a factor of 1.5). A decrease of the R2/R1 ratio from 2.5 to 2 allows an increase in the electric field and hence the detected frequency in addition to the scaling factor, thus bringing the total gain to about 1.8-fold. The smaller cell required an increase of the injection ion energy of about 1.4-fold for the same 5000-V voltage on the central electrode. Despite the increase in space charge density in the analyzer by a factor of (1.5)^3^ ≈ 3.4, the additional shielding provided by the relatively thicker central electrode keeps space-charge-induced frequency shifts even slightly below those in the standard analyzer (27). A miniature electrostatic lens provides sharp spatial focusing of ions coming into the injection slot located on one of the injection electrodes. As ion packets from the C-trap enter the analyzer off-axis, axial oscillations are initiated without the need for any additional excitation and without a loss of synchronization with the moment of ejection from the C-trap. This facilitates the use of enhanced Fourier transform (28) for all modes of operation in the same way as in the preceding Q Exactive instrument.

The Q Exactive HF is preset to record spectral data at five distinct resolutions, namely, 15,000, 30,000, 60,000, 120,000, and 240,000 at *m*/*z* 200 Th. Each resolution corresponds to a transient time denoting the time spent on analyzing the ion population in the Orbitrap mass analyzer, respectively consisting of 32, 64, 128, 256, and 512 ms. These resolutions are slightly reduced for the ultra-high-field analyzer relative to the equivalent settings for the classic analyzer, as the total gain of the resolution at the same transient time is a factor of 1.8 rather than 2. The Q Exactive HF instrument additionally makes a higher maximum resolution of 240,000 available. By comparing the time difference between each consecutive scan for the ultra-high-field Orbitrap analyzer to the theoretical values for the transient times, we found that the overhead for each scan was between 6 and 14 ms (Fig. 4*B*). This constitutes an improvement over the 17-ms scan time overhead for the Q Exactive. When the increased transient speed is utilized to double the number of scans at roughly the same resolution, this reduction of the scan time overhead is required to keep the injection times at a reasonably high level when the instrument is running in fully parallel mode. For instance, for the 15,000 resolution setting the transient time is reduced from 64 to 32 ms; at a scan time overhead of 17 ms, more than half of the available time for the scan would have been lost without minimizing overhead. For shotgun analysis of complex mixtures, running in full parallel mode is desirable in terms of peptide identifications. We found the 15,000 resolution setting (corresponding to 32-ms transients) in combination with a maximum injection time of 25 ms to be best for complex mixtures such as total cell lysates (supplemental Fig. S3). To investigate the effect of the minor nominal reduction of instrument resolution, we measured the actual resolution of the identified peaks of a whole HeLa cell lysate. A slight drop in the resolution of those peaks was indeed observed (Fig. 4*C*); however, this is a minor effect, and we did not notice any impact on downstream data analysis. As described below, the increased scan speed indeed resulted in significantly increased numbers of peptide and protein identifications (Fig. 4*D*). We also note that one can double the MS resolution for the Q Exactive HF relative to that of the Q Exactive (120,000 *versus* 70,000) without detrimental consequences. This is because the transient time gained through acquisition at the lower resolution would be only 128 ms, which at 10% is an insignificant part of the total cycle.

##### Optimal Parameter Value Verification

After individually optimizing the parameters for the Q Exactive HF, we performed an independent verification of the optimal values for each of the parameters using a design-of-experiment approach (29, 30) using MODDE (Umetrics, Frankfurt, Germany). A benefit of such an approach is that it gives insight into parameters that have potentially interacting properties. For the analysis we chose the parameters that previously gave the greatest effect in our experiments, consisting of normalized collision energy (20–40), S-lens rf level (40–80), and isolation window (0.4–3 Th). The range of the parameters was chosen to be large and center on the previously found optimal values. All RAW data were recorded based on the experimental design established by the software. From the results of this study we concluded that the previously described values are optimal and that in our set there were no significantly interacting parameters present from which a performance benefit could be gained. As the conclusion remained the same and a design-of-experiment approach can deal with sparse data, this can be an attractive study type for quickly optimizing LC-MS/MS metrics for different types of samples and chromatography separation (supplemental Fig. S10).

##### Q Exactive Performance for Total Cell Lysate Analysis

**Fig. 5.**
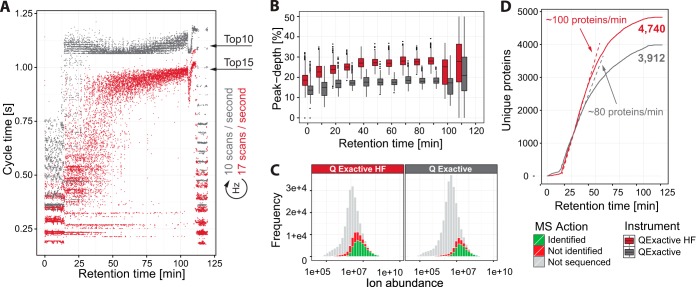
**Performance of the Q Exactive HF with ultra-high-field Orbitrap analyzer.** *A*, cycle-time comparison for the Q Exactive and the Q Exactive HF on a complex HeLa total cell lysate. *B*, sequencing peak-depth comparison between the Q Exactive and the Q Exactive HF. *C*, comparison of the total number of visible peptides (cumulative green, red, and gray), sequenced peptides (cumulative green and red), and successfully identified peptides (green) between the Q Exactive and the Q Exactive HF. *D*, cumulatively identified proteins over the gradient comparison between the Q Exactive and the Q Exactive HF. The dotted lines denote a linear fit on the steepest part of the curve.

We next directly compared the Q Exactive HF to the Q Exactive using the standard top-10 method on a 2 h gradient for the Q Exactive, which has demonstrated effectiveness in addressing a wide variety of biological questions. We ensured a fair comparison by running the test within one day, using the same sample, HPLC, and analytical column on both systems. We wished to make use of the increased speed of the Q Exactive HF while still achieving a cycle time of around 1 s and occupying the instrument with close to the maximum number of fragmentation scans in each cycle. For this, we found a top-15 method to be the most effective. An example of the MS spectrum with the subsequent fragmentation spectra can be seen in supplemental Fig. S1. In this comparison, we achieved cycle times of 1.1 s on the Q Exactive and 0.9 s on the Q Exactive HF. The extra five scans per cycle at roughly the same cycle time translate into a speed increase from 10 scans per second to 17 scans per second (Fig. 5*A*). In a direct comparison between the Q Exactive and the Q Exactive HF, we found that the higher speed delivered an increase of almost 60% more scans (variation over five runs ± 620 scans). At an identification success rate of 62% in both cases, this translated into 48% more MS2 identifications (variation over five runs ± 313 identifications) and 43% more unique sequences (variation over five runs ± 238 sequences) (Fig. 4*D*).

One prominent feature of the cycle-time plot is that the Q Exactive reached the full top *N* early on in the gradient, whereas the Q Exactive HF achieved the full top *N* much later in the gradient. We interpret this to mean that even in highly complex samples such as HeLa full cell lysates, the initial part of the gradient does not contain sufficient precursor ions that fulfill the criteria for selection for fragmentation at these very high sequencing speeds.

To investigate and compare the impact of the extra sequencing speed, we determined the achieved peak depth for each of the instruments. Here we define this value as the position of the precursor in the list of all visible precursors in the cycle where it is sequenced, sorted on descending ion abundance. For complex HeLa digests, we found that this list contained around 400 precursors (distilled from around 4000 individual peaks in the spectrum) at any given time during the linear part of the gradient (supplemental Fig. S7). The Q Exactive probed this list to a medium depth of 18%, or around 72 precursors per cycle, whereas the Q Exactive HF, with its enhanced speed, achieved a depth of 27%, or around 108 isotopes per cycle (Fig. 5*B*). This additional peak depth was indeed translated into more fragmented precursors, which were increased by 53% from 48,449 to 74,383. As both instruments exhibited a sequencing success rate of more than 50% over the entire gradient, this translated into an increase of 43%, from 27,256 to 39,119 unique peptide sequences (Fig. 5*C*). There was a slight reduction in the number of visible peptides (cumulative green, red, and gray population), which we attribute to the lower resolution of the full scans for the Q Exactive HF. From the histogram, however, it is evident that the instrument sequenced down to lower abundance peptides (cumulative green and red population) and is capable of successfully identifying a greater proportion of available peptides (green population). These additionally sequenced peptides contribute to new protein identifications, as opposed to only extending the sequence coverage of already accessed proteins. This is evident from a 21% increase in identified proteins, from 3912 to 4740. Thus, the sequencing speed enabled by the ultra-high-field Orbitrap analyzer allows identification of a greater percentage of total detectable precursors, but a majority of unfragmented peptides remains. This is not entirely due to their low abundance, as many peptides in the gray population have a signal similar to those of fragmented and successfully identified peptides. This suggests that further increases in sequencing speed would still be useful for particularly congested parts of the gradient. Plotting the cumulative number of proteins over the gradient revealed that the Q Exactive HF improved the maximum protein sequencing rate by 25%, from 80 to 100 proteins per minute (Fig. 5*D*).

Further investigations into the dynamic range of the identified peptides revealed that both instruments reliably sequenced over 3 orders of magnitude, indicating that the performance increase of the Q Exactive HF can be attributed to its ability to sequence more peptides in the busy regions of chromatography. This additional speed is also responsible for a higher inter-replicate reproducibility at the peptide identification level, where the instrument achieves better reproducibility for lower peptide abundances (supplemental Fig. S8). To investigate the Q Exactive HF performance on different gradient lengths and to provide an indication of optimal performance for the instrument, we additionally ran a gradient titration series on the standard HeLa samples, which revealed that gradients over 4 h did not further improve identification performance for our sample. Based on the increase per time unit, we concluded that a 150-min gradient represents the optimal length. The trend in our and other laboratories to measure over 4-h or longer gradients can now to some extent be reversed (supplemental Fig. S9).

**Fig. 6.**
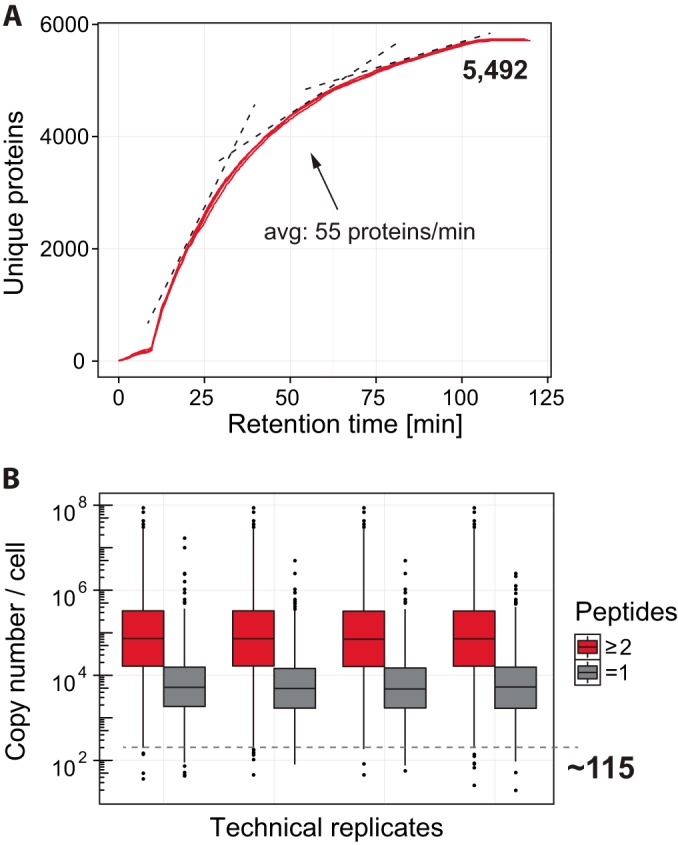
**Optimized chromatography performance.** *A*, cumulative proteins over the gradient. *B*, copy numbers per cell for the detected proteins.

As we observed that the Q Exactive HF did not routinely achieve the full top *N*, we hypothesized that the peak depth was limited by chromatography rather than the sequencing speed of the mass spectrometer. To address this question, we employed DMSO as a dopant in the mobile phase buffers, as it had been reported to significantly increase the ion flux and consequently the performance of shotgun proteomics experiments (31). On the standard 2 h gradient, peptide identifications increased by 13%, from 39,119 to 44,446, and protein identification increased by 16%, from 4740 to 5492 (Fig. 6*A*). Even with this addition of potentially sequenceable peptides, the instrument still did not reach the full top *N* per cycle, meaning that further increases in ion abundance would be beneficial. The achieved protein sequencing rate over the actual gradient (subtracting the lead-in time of 5 min for the buffers to arrive and a washout phase of 15 min) was 55 proteins per minute for these optimized conditions. When comparing the copy numbers for HeLa cells to the detected proteins (32), we found that the instrument was capable of successfully sequencing in an estimated protein dynamic range from 90 million down to ∼115 copies per cell (Fig. 6*B*), enabling the analysis of highly complex samples down into the transcription factor range in reasonable time. Although the dimethyl sulfoxide dopant in our experiments had clear benefits in terms of performance, we do not routinely use it because it represents a potential contamination source for the quadrupole rods, which cannot be resolved through the incorporation of the selecting inject flatapole. Further studies are clearly needed to confirm its effects on instrument robustness.

##### Application to Phosphoproteomics

**Fig. 7.**
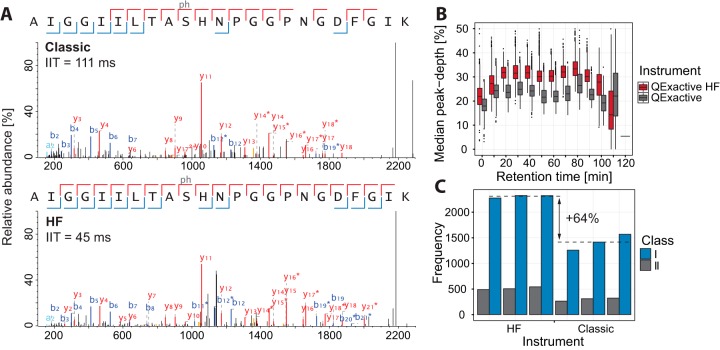
**Phosphorylation enriched samples.** *A*, example fragmentation spectra of the same phosphorylated peptide identified on the Q Exactive (*top*) and on the Q Exactive HF (*bottom*). In both cases the maximum allowed ion injection time was reached. *B*, comparison of the achieved peak-depth on the Q Exactive and the Q Exactive HF. *C*, comparison of the successfully sequenced Class I phosphorylation sites.

Given the observed increase in performance for the whole cell lysates, we reasoned that the Q Exactive HF might be capable of improved performance on sample types requiring high mass spectrometric sensitivity as well. In order to test this, we measured an unstimulated HeLa lysate enriched for phosphorylated peptides with both instruments. Phosphopeptides are generally of low abundance, and therefore it is advantageous to allow for longer ion injection times for the fragmentation scans to accumulate a sufficient amount of ions for a successful sequencing event (33). Here we chose a maximum of 111 ms for the Q Exactive and 45 ms for the Q Exactive HF, which took the 17-ms scan time overhead of the original Q Exactive into account (leaving the Q Exactive HF at a slight disadvantage). Fig. 7*A* illustrates the analysis on both instruments for the same phosphorylated peptide. Even though in both cases the maximum allowed ion injection time was reached—meaning that not enough ions were present to fulfill the request of 1e5 ions for the higher energy collisional dissociation scan—almost full sequence coverage was achieved in both cases. The Q Exactive HF, however, achieved this coverage in less than half the ion injection time. We investigated whether the automatic gain control target could still be achieved at these restricted maximum injection times for all the MS2 scans and found that it could in the majority of cases for both instruments. As in the above example, most of the MS2 events for the classic instrument required almost double the time in this experiment (supplemental Fig. S11). The longer ion injection time in the sensitive method already allows the Q Exactive to probe deeper into the list of visible precursors, from a median of 18% for the normal method to 23% for the sensitive method. However, the additional speed of the Q Exactive HF allowed it to go even further, from a median of 27% for the normal method to 33% for the sensitive method (Fig. 7*B*). Overall, the increased sequencing speed in combination with the improved quadrupole transmission translated into 64% more class I phosphorylation sites identified in a single run in this particular experimental situation (Fig. 7*C*). However, in experimental situations where the phosphopeptide amounts necessitated longer fill times, we have observed much smaller gains.

##### Conclusions and Outlook

Here we have described the next iteration of the Exactive family of mass spectrometers, the Q Exactive Plus and the Q Exactive HF, and investigated its performance on analytical standards and on complex peptide mixtures for shotgun proteomics using our standard 90-min gradient. The ultra-high-field Orbitrap analyzer doubles the sequencing speed at the same resolution, for which the improved ion transmission characteristics of the segmented quadrupole at least partially provide the necessary increase in precursor ion abundance. The higher speed translates well to actual shotgun proteomics improvements, as we observed increases of more than 40% in unique peptide sequences and more than 20% in proteins relative to the previous generation on our standard gradient with the HeLa cell lysate.

We observed that the mass spectrometer did not fully make use of its potential sequencing speed, as there were insufficient ions in parts of the gradient to satisfy the requirements for attempting a sequencing event. In order for this to be resolved, optimizations such as dopant-enhanced mobile phases (31), higher sensitivity analytical columns (34), or brighter ion sources (35) will be required to increase the precursor ion abundance. In applications with very complex samples containing a sufficient number of sequenceable precursors, we anticipate that researchers will be able to boost the instrument productivity almost 2-fold while still achieving the same sequencing depth as before. With such a large reduction in analysis time, any overhead between LC-MS/MS runs becomes increasingly undesirable. For example, currently the loading times on our single analytical column setup (50 cm; C_18_) are around 30 min. During this time the mass spectrometer is not recording useful data, which is still acceptable for an analysis time of 2 h given that this loss corresponds to two runs per day. At a 1-h analysis time, however, an overhead of 30 min results in a loss of almost eight runs per day. Thus optimized chromatography setups, such as pre-columns that do not degrade resolution or double analytical columns, will be needed in order for the instrument to be used most effectively.

In conclusion, this new generation of quadrupole Orbitrap instruments is designed for significantly greater robustness, and our experiments confirmed that the majority of undesired ions were confined to the front part of the instrument. We likewise observed significantly enhanced selection characteristics of the new segmented quadrupole, which should be useful in many proteomic experiments. Finally, and most important, the ultra-high-field Orbitrap analyzer in the Q Exactive HF routinely provided doubled resolution in MS scans without downsides and doubled the potential sequencing speed in MS/MS mode.

## Supplementary Material

Supplemental Data

## Footnotes

^1^ The abbreviations used are:

MS: mass spectrometry
MS/MS: tandem mass spectrometry
ACN: acetonitrile.
