# Supplementary material for: The Q Exactive HF, a Benchtop Mass Spectrometer with a Pre-filter, High-performance Quadrupole and an Ultra-high-field Orbitrap Analyzer
Source: Mol Cell Proteomics. 2014 Oct 30;13(12):3698–708. doi: 10.1074/mcp.M114.043489 (PMC4256516; doi:10.1074/mcp.M114.043489)
Supplement: Supplemental Data [file supp_13_12_3698__index.html]

The Q Exactive HF, a Benchtop Mass Spectrometer with a Pre-filter, High-performance Quadrupole and an Ultra-high-field Orbitrap Analyzer — Q Exactive with Ultra-high-field Orbitrap Analyzer — Supplemental Data 

# The Q Exactive HF, a Benchtop Mass Spectrometer with a Pre-filter, High-performance Quadrupole and an Ultra-high-field Orbitrap Analyzer

## Supplemental Data

**Files in this Data Supplement:**

- Online suppl material - Online suppl material containing suppl tables and figures
- MQ protein group list - MQ protein group list. Excel list of identified proteins.
- High res Figures and Suppl Figures - High res Figures and Suppl Figures (same as in suppl material, only high resolution)
