# Supplementary material for: The Q Exactive HF, a Benchtop Mass Spectrometer with a Pre-filter, High-performance Quadrupole and an Ultra-high-field Orbitrap Analyzer
Source: Mol Cell Proteomics. 2014 Oct 30;13(12):3698–708. doi: 10.1074/mcp.M114.043489 (PMC4256516; doi:10.1074/mcp.M114.043489)
Supplement: Supplemental Data [file supp_M114.043489_hf-supplementary.pdf]

Supplementary table 1

Experiment type	Comments
Calmix direct infusion	Direct infusion of Thermo Velos Calmix solution electrosprayed with an ESI source and delivered from a 500 ml Hamilton syringe at 5 μl / min.
Q Exactive HF	Top15 shotgun experiment on the Q Exactive Plus with Orbitrap ultra-high field analyzer. A sample of 2 μg HeLa peptides electrosprayed with the nano-ESI source and delivered over a 50 cm reversed phase C18 analytical column by a Thermo Easy ultra-nLC.<br><br>Unless otherwise stated, the gradient was 2hr (90 min linear).
Q Exactive	Top10 shotgun experiment on the Q Exactive with standard Orbitrap analyzer. A sample of 2 μg HeLa peptides electrosprayed with the nano-ESI source and delivered over a 50 cm reversed phase C18 analytical column by a Thermo Easy ultra-nLC.<br><br>Unless otherwise stated, the gradient was 2hr (90 min linear).
MaxQuant	The complete txt-folder forming the MaxQuant results.
Andromeda	The complete low-level output from the Andromeda search engine. This allows one, combined with the msms.txt from the MaxQuant results, to view the annotated MS/MS spectra in ‘Protein Prospector MS-Viewer’.

Supplementary table 2

	Associated files	Experiment type	Panels	Comments
**Figure 2**	20140228_RiSc_QC_calmix_prefilter.raw	Calmix direct infusion	**A**, **B**.	Spectra extracted with Xcalibur (Edit → Copy special).<br><br>Intensity values for specific m/z values extracted with MSFileReader (option centroid 'on').<br><br>Used scans 102 and 1808.
	20140129_EXQ00_RiSc_SA_BREMENHELA_UF1_01.raw	Q Exactive HF	**C**.	Precursor m/z and spectral data extracted with MSFileReader.
**Figure 3**	20140113_RiSc_SA_calmix_isolation.raw	Calmix direct infusion	**A**.	Intensity values for specific m/z values extracted with MSFileReader (option centroid 'on').
	-	Tune output.	**B**.	
	20131221_EXQ00_RiSc_QC_HELA_iso0_4.raw<br>20131221_EXQ00_RiSc_QC_HELA_iso0_6.raw<br>20131221_EXQ00_RiSc_QC_HELA_iso0_8.raw<br>20131221_EXQ00_RiSc_QC_HELA_iso1_0.raw<br>20131221_EXQ00_RiSc_QC_HELA_iso1_2.raw<br>20131221_EXQ00_RiSc_QC_HELA_iso1_4.raw<br>20131221_EXQ00_RiSc_QC_HELA_iso1_6.raw<br>20131221_EXQ00_RiSc_QC_HELA_iso1_8.raw<br>20131221_EXQ00_RiSc_QC_HELA_iso2_0.raw<br>20131221_EXQ00_RiSc_QC_HELA_iso2_2.raw<br>20131221_EXQ00_RiSc_QC_HELA_iso2_4.raw	Q Exactive HF	**C**, **D**.	Standard settings were used with varying isolation window (denoted in the filename).<br><br>MaxQuant output 'Peptide Sequences Identified' from the file 'summary.txt'; maximum scaled to 100%.
**Figure 4**	20140228_RiSc_QC_calmix_transient_02.raw	Calmix direct infusion	**B**.	Retention times extracted with MSFileReader.
	20140129_EXQ00_RiSc_SA_BREMENHELA_UF1_01.raw<br>20140130_EXQ5_RiSc_SA_BREMEN_HELA_01.raw	Q Exactive HF<br>Q Exactive	**C**, **D**.	Resolution was measured by MaxQuant. Values found in the file 'allPeptides.txt'.
**Figure 5**	20140129_EXQ00_RiSc_SA_BREMENHELA_UF1_01.raw<br>20140129_EXQ00_RiSc_SA_BREMENHELA_UF1_02.raw<br>20140129_EXQ00_RiSc_SA_BREMENHELA_UF1_03.raw<br>20140129_EXQ00_RiSc_SA_BREMENHELA_UF1_04.raw<br>20140129_EXQ00_RiSc_SA_BREMENHELA_UF1_05.raw<br>20140130_EXQ5_RiSc_SA_BREMEN_HELA_01.raw<br>20140130_EXQ5_RiSc_SA_BREMEN_HELA_02.raw<br>20140130_EXQ5_RiSc_SA_BREMEN_HELA_03.raw<br>20140130_EXQ5_RiSc_SA_BREMEN_HELA_04.raw<br>20140130_EXQ5_RiSc_SA_BREMEN_HELA_05.raw<br><br>qcompare.zip<br>qcompare-andromeda.zip	Q Exactive HF<br><br><br><br><br>Q Exactive<br><br><br><br><br><br>MaxQuant<br>Andromeda	**A**, **B**, **C**, **D**.	Each file was processed by MaxQuant as an individual experiment. This causes the proteinGroups.txt to contain a separate intensity entry for each protein-group. When intensity > 0, the protein was counted.
**Figure 6**	20140201_EXQ00_RiSc_SA_STEVENHELA_01.raw<br>20140201_EXQ00_RiSc_SA_STEVENHELA_02.raw<br>20140201_EXQ00_RiSc_SA_STEVENHELA_03.raw<br>20140201_EXQ00_RiSc_SA_STEVENHELA_04.raw<br>20140201_EXQ00_RiSc_SA_STEVENHELA_60min_01.raw	Q Exactive HF	**A**, **B**.	

	20140201_EXQ00_RiSc_SA_STEVENHELA_60min_02.raw<br>20140201_EXQ00_RiSc_SA_STEVENHELA_60min_03.raw<br>20140201_EXQ00_RiSc_SA_STEVENHELA_60min_04.raw			
**Figure 7**	20140116_EXQ00_RiSc_SA_IGORPH_02_lowres.raw<br>20140116_EXQ00_RiSc_SA_IGORPH_03_lowres.raw<br>20140116_EXQ00_RiSc_SA_IGORPH_04_lowres.raw<br>20140124_EXQ5_KoMa_QC_HeLa_phos_120min_50cm2.raw<br>20140124_EXQ5_KoMa_QC_HeLa_phos_120min_50cm3.raw<br>20140124_EXQ5_KoMa_QC_HeLa_phos_120min_50cm4.raw<br><br>qcompare-phospho.zip<br>qcompare-phospho-andromeda.zip	Q Exactive HF<br><br><br>Q Exactive<br><br><br><br>MaxQuant<br>Andromeda	**A**, **B**.	Annotated spectra extracted with the MQ viewer.<br><br>The Q Exactive+ runs were run at an MS2 resolution of 15k because of a hardware occurring at 30k.
**Suppl. Figure 1**	20140129_EXQ00_RiSc_SA_BREMENHELA_UF1_01.raw	Q Exactive HF	-	The raw-file was processed individually with MaxQuant option 'Calculate peak properties' turned on.
**Suppl. Figure 2**	20140110_EXQ00_RiSc_QC_HELA_nce25.raw<br>20140110_EXQ00_RiSc_QC_HELA_nce26.raw<br>20140110_EXQ00_RiSc_QC_HELA_nce27.raw<br>20140110_EXQ00_RiSc_QC_HELA_nce28.raw<br>20140110_EXQ00_RiSc_QC_HELA_nce29.raw<br>20140110_EXQ00_RiSc_QC_HELA_nce30.raw	Q Exactive HF	**A**, **B**, **C**.	Standard settings were used with varying Normalized Collision Energy (NCE; denoted in the filename).<br><br>1hr gradient.
**Suppl. Figure 3**	20140126_EXQ00_RiSc_SA_BREMENHELA_20ms.raw<br>20140126_EXQ00_RiSc_SA_BREMENHELA_25ms.raw<br>20140126_EXQ00_RiSc_SA_BREMENHELA_30ms.raw<br>20140126_EXQ00_RiSc_SA_BREMENHELA_35ms.raw<br>20140126_EXQ00_RiSc_SA_BREMENHELA_40ms.raw<br>20140126_EXQ00_RiSc_SA_BREMENHELA_45ms.raw<br>20140126_EXQ00_RiSc_SA_BREMENHELA_50ms.raw<br>20140126_EXQ00_RiSc_SA_BREMENHELA_55ms.raw<br>20140126_EXQ00_RiSc_SA_BREMENHELA_60ms.raw	Q Exactive HF	**A**, **B**, **C**, **D**.	Standard settings were used with varying Ion Injection Time (IIT; denoted in the filename).
**Suppl. Figure 4**	20131221_EXQ00_RiSc_QC_HELA_rf45.raw<br>20131221_EXQ00_RiSc_QC_HELA_rf50.raw<br>20131221_EXQ00_RiSc_QC_HELA_rf55.raw<br>20131221_EXQ00_RiSc_QC_HELA_rf60.raw<br>20131221_EXQ00_RiSc_QC_HELA_rf65.raw<br>20131221_EXQ00_RiSc_QC_HELA_rf70.raw<br><br>mq-output-tryptic.zip<br>mq-output-unspecific.zip	Q Exactive HF<br><br><br><br><br><br><br>MaxQuant	**A**, **B**, **C**.	Standard settings were used with varying s-lens RF level (denoted in the filename).<br><br>1hr gradient.
**Suppl. Figure 5**	20140110_EXQ00_RiSc_QC_HELA_uf020.raw<br>20140110_EXQ00_RiSc_QC_HELA_uf040.raw<br>20140110_EXQ00_RiSc_QC_HELA_uf060.raw<br>20140110_EXQ00_RiSc_QC_HELA_uf080.raw<br>20140110_EXQ00_RiSc_QC_HELA_uf100.raw	Q Exactive HF	**A**, **B**.	Standard settings were used with varying underfil ratio values (denoted in the filename).<br><br>1hr gradient.
**Suppl. Figure 6**	20140421_EXQ00_RiSc_QC_HELA_target02e4.raw<br>20140421_EXQ00_RiSc_QC_HELA_target05e4.raw<br>20140421_EXQ00_RiSc_QC_HELA_target10e4.raw<br>20140421_EXQ00_RiSc_QC_HELA_target20e4.raw	Q Exactive HF		Standard settings were used with varying MS2 target values (denoted in the filename).<br><br>1hr gradient.
**Suppl. Figure 7**	20140129_EXQ00_RiSc_SA_BREMENHELA_UF1_01.raw<br>20140130_EXQ5_RiSc_SA_BREMEN_HELA_01.raw	Q Exactive HF<br>Q Exactive	**A**, **B**.	Data was extracted from the

				msScans.txt, msmsScans.txt, and allIsotopes.txt tables exported by MaxQuant.
**Suppl. Figure 8**	20140129_EXQ00_RiSc_SA_BREMENHELA_UF1_01.raw<br>20140129_EXQ00_RiSc_SA_BREMENHELA_UF1_02.raw<br>20140129_EXQ00_RiSc_SA_BREMENHELA_UF1_03.raw<br>20140129_EXQ00_RiSc_SA_BREMENHELA_UF1_04.raw<br>20140129_EXQ00_RiSc_SA_BREMENHELA_UF1_05.raw<br>20140130_EXQ5_RiSc_SA_BREMEN_HELA_01.raw<br>20140130_EXQ5_RiSc_SA_BREMEN_HELA_02.raw<br>20140130_EXQ5_RiSc_SA_BREMEN_HELA_03.raw<br>20140130_EXQ5_RiSc_SA_BREMEN_HELA_04.raw<br>20140130_EXQ5_RiSc_SA_BREMEN_HELA_05.raw	Q Exactive HF<br><br><br><br><br>Q Exactive	**A**, **B**.	MaxQuant output as described for. Figure 5 was used.
**Suppl. Figure 9**	20140924_EXQ00_RiSc_SA_BremenHeLa_060min.raw<br>20140924_EXQ00_RiSc_SA_BremenHeLa_090min.raw<br>20140924_EXQ00_RiSc_SA_BremenHeLa_120min.raw<br>20140924_EXQ00_RiSc_SA_BremenHeLa_150min.raw<br>20140924_EXQ00_RiSc_SA_BremenHeLa_180min.raw<br>20140924_EXQ00_RiSc_SA_BremenHeLa_210min.raw<br>20140924_EXQ00_RiSc_SA_BremenHeLa_240min.raw	Q Exactive HF	**A**, **B**, **C**.	60 to 240 min gradient in steps of 30 minutes
**Suppl. Figure 10**	20140825_EXQ00_DaHo_SA_BremenHeLa_01_04_80_20<br>20140825_EXQ00_DaHo_SA_BremenHeLa_02_04_40_40<br>20140825_EXQ00_DaHo_SA_BremenHeLa_03_04_60_30<br>20140825_EXQ00_DaHo_SA_BremenHeLa_04_17_60_30<br>20140825_EXQ00_DaHo_SA_BremenHeLa_05_17_60_40<br>20140825_EXQ00_DaHo_SA_BremenHeLa_06_17_60_30<br>20140825_EXQ00_DaHo_SA_BremenHeLa_07_17_60_20<br>20140825_EXQ00_DaHo_SA_BremenHeLa_08_17_80_30<br>20140825_EXQ00_DaHo_SA_BremenHeLa_09_3_40_40<br>20140825_EXQ00_DaHo_SA_BremenHeLa_10_3_40_20<br>20140825_EXQ00_DaHo_SA_BremenHeLa_11_04_40_20<br>20140825_EXQ00_DaHo_SA_BremenHeLa_12_17_40_30<br>20140825_EXQ00_DaHo_SA_BremenHeLa_13_3_80_20<br>20140825_EXQ00_DaHo_SA_BremenHeLa_14_3_60_30<br>20140825_EXQ00_DaHo_SA_BremenHeLa_15_04_80_40<br>20140825_EXQ00_DaHo_SA_BremenHeLa_16_17_60_30<br>20140825_EXQ00_DaHo_SA_BremenHeLa_17_3_80_40	Q Exactive HF	**A**, **B**, **C**, **D**.	1h gradient.
**Suppl. Figure 11**	20140116_EXQ00_RiSc_SA_IGORPH_02_lowres.raw<br>20140116_EXQ00_RiSc_SA_IGORPH_03_lowres.raw<br>20140116_EXQ00_RiSc_SA_IGORPH_04_lowres.raw<br>20140124_EXQ5_KoMa_QC_HeLa_phos_120min_50cm2.raw<br>20140124_EXQ5_KoMa_QC_HeLa_phos_120min_50cm3.raw<br>20140124_EXQ5_KoMa_QC_HeLa_phos_120min_50cm4.raw	Q Exactive HF<br><br><br>Q Exactive	**A**, **B**.	MaxQuant output as described for. Figure 7 was used.
**Further data**	20140317_EXQ00_RiSc_SA_HeLa_60min_top27_01<br>20140317_EXQ00_RiSc_SA_HeLa_60min_top27_02<br>20140317_EXQ00_RiSc_SA_HeLa_60min_top27_03	Q Exactive HF		Fast runs with a top 27 method

Supplementary table 3

Setting	QExactive	QExactive HF
Instrument		
S-Lens RF-level	60	60
Capillary temperature	250 °C	250 °C
Full scan		
Microscans	1	1
Resolution	70.000	60.000
AGC target	3e6	3e6
Maximum IT	20 ms	20 ms
Scan range	300 - 1650 m/z	300 - 1650 m/z
dd-MS2		
Microscans	1	1
Resolution	17.500	15.000
AGC target	1e5	1e5
Maximum IT	120 ms	25 ms
TopN (no msx)	10	15
Isolation window	3	1.4 m/z
Isolation offset	-	0.0 m/z
Fixed first mass	Off	Off
NCE (no stepped NCE)	25	27
Apex trigger	Off	Off
Charge exclusion	Unassigned, 1	Unassigned, 1
Peptide match	On	Off
Exclude isotopes	On	On
Dynamic exclusion	20s	20s

Mass spectrometry related settings used for the instrument comparison. We varied the values for parameters relevant for the best performance of each instrument. These include: resolution (due to the compromise of slightly lower resolution for the same transient time), maximum injection time (due to the faster transient), NCE (due to a different HCD energy scaling function) and peptide match (due to the instrument not achieving full topN).

Supplementary Figure 1

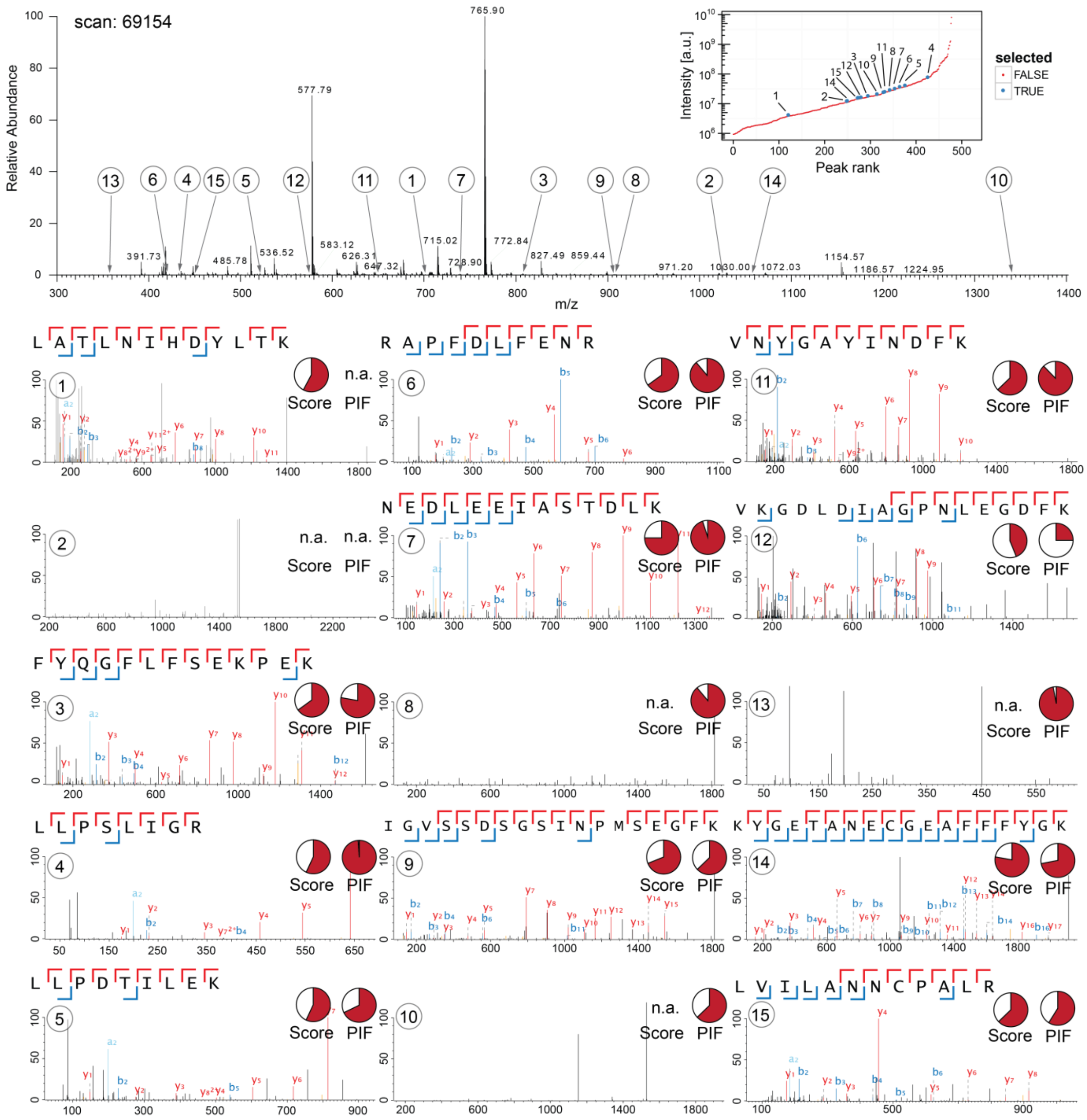

Performance of the Q Exactive HF on a single cycle with identification rate of over 70% at a speed of 17 Hz. Top panel represents the full scan preceding the fragmentation scans, where the isotope used for each fragmentation scan is marked with an arrow and number indicating its position in the cycle. In the inset the isotopes are ranked on intensity with the location of the sequenced ones marked, showing the instrument is sequencing over an order of magnitude within a single cycle. The annotated fragmentation spectra in this cycle are presented below. The first two sequencing events are of lower intensity, which can be attributed to the high sequencing speed and the software catching up with the most up-to-date information. The pie-charts indicate the Andromeda Score associated to the identification on log2 scale and the Precursor Ion Fraction (PIF; also isolation purity) of the fragmentation spectrum.

Supplementary Figure 2

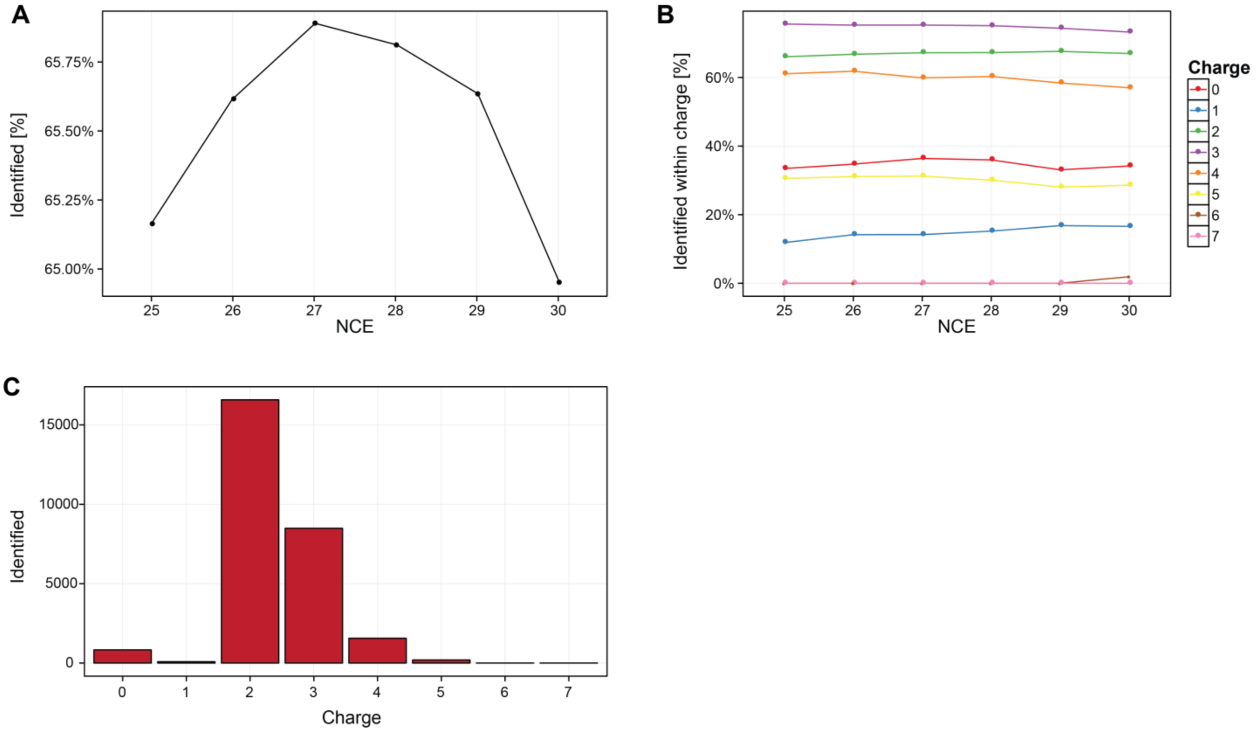

Normalized Collision Energy (NCE) titration series. Data collected with the standard settings (Xcalibur was configured to exclude charge-state unassigned, 1, and 6 and up), while varying the NCE, on a HeLa whole cell lysate (see Supplementary Table 2 for further description). The range was chosen based on experience with the Q Exactive, where the optimal value was found to be 25 (data not shown), and the expectation a higher value was required due to a change in the NCE to CE scaling function. **A.** Optimal overall performance, within a small variation, was found at NCE level of 27 (setting used for further experiments). **B.** Success-rate within charge-states (0 denotes a single peak for which no charge state could be determined). Even though both unassigned and charge-state 1 are excluded, these are still sequenced as the control software has to decide the charge state based on not yet complete data. The identification success-rate of charge-state 5 is generally low, while charge-states 2, 3, and 4 have over 60% success-rate. **C.** Higher charge-states are excluded, given the minimal contribution from charge-state 5 to the overall identifications (here at NCE 27), potentially creating a better focus for the mass spectrometer.

Supplementary Figure 3

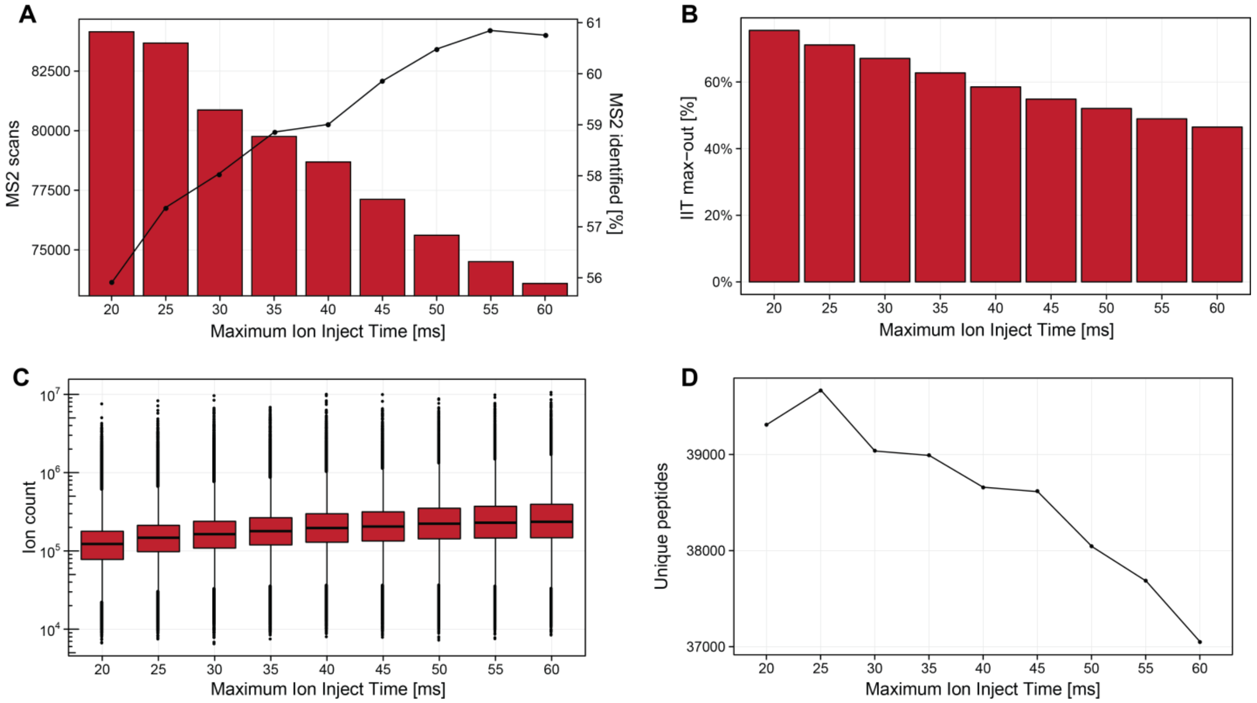

Ion Injection Time (IIT) titration series. Data collected with the standard settings, while varying the maximum allowed IIT, on a HeLa whole cell lysate (see Supplementary Table 2 for further description). The range was chosen to encompass the speed of the Orbitrap ultra-high field analyzer running at 15k resolution for the MS2 scans. **A.** With longer maximum IIT's the instrument is achieving less MS2 scans (bars in the graph), as the MS2 scan fill time does not fit in the transient time of the previous MS2 scan (i.e. parallel mode). Below 25 ms there is only a slight increase in performed MS2 scans. With the longer IIT's the sequencing success-rate is however increasing (line in the graph). **B.** That the success-rate is increasing can be attributed to the longer allowed IIT, causing less MS2 scans actually to reach the maximum IIT value (i.e. max-out). **C.** This ensures that the full ion population of 1e5 is reached in more cases. **D.** The optimal IIT for the MS2 scans is however found around 25 ms (plus the reported median 6 to 14 ms scan time overhead makes this value in a lot of cases fully parallel with the previous MS2 scan). Even with the almost 70% max-out rate and consequently lowered sequencing success-rate at this IIT, the parallel operation ensures that many more MS2 scans can be performed making up for the difference. The lowered performance at 20 ms is expected, as at 25 ms most of the MS2 scans were already in parallel mode and the further lowered sequencing success rate is not making up for the extra MS2 scans being performed.

Supplementary Figure 4

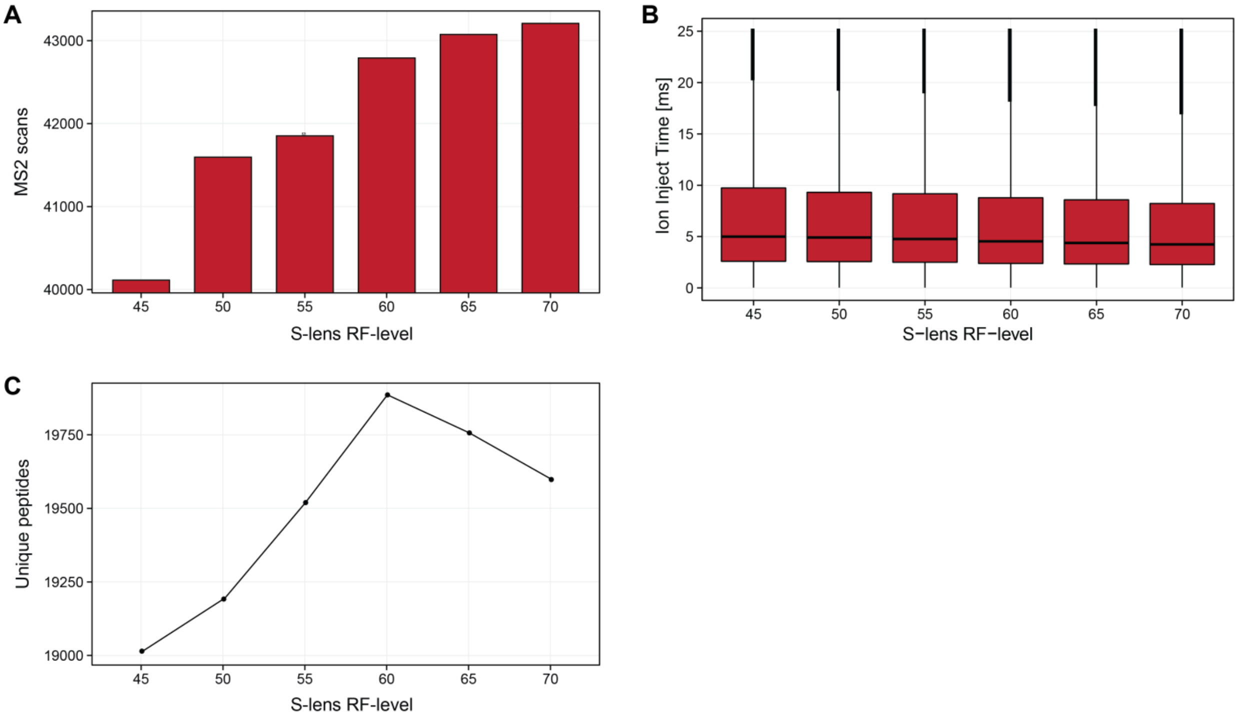

S-lens RF-level titration series. Data collected with the standard settings, while varying the S-lens RF-level, on a HeLa whole cell lysate (see Supplementary Table 2 for further description). The chosen value for the S-lens RF-level affects the transmission through the S-lens. **A.** The increase in transmission for the lower m/z region at higher S-lens RF-levels has the effect that more isotopes will pass the minimal ion threshold required, resulting in more fragmentation scans being performed. **B.** The increased ion transmission also results in lower IIT's for MS2 scans (only MS2 scans presented not reaching the maximum IIT). **C.** We find optimal performance at the S-lens RF-level of 60, where most unique peptide sequences are reported. Of concern with the S-lens RF-levels is that source fragmentation is more likely to occur at higher values, resulting in isotope patterns for non-tryptic peptides that cannot be identified. This most probably happens for S-lens RF-level 65 and 70, where fewer unique peptide sequences are reported. At the used S-lens levels we found however that any fragmentation results in hard to detect levels, as an unspecific search of the fragmentation spectra did not yield any non-tryptic peptides.

Supplementary Figure 5

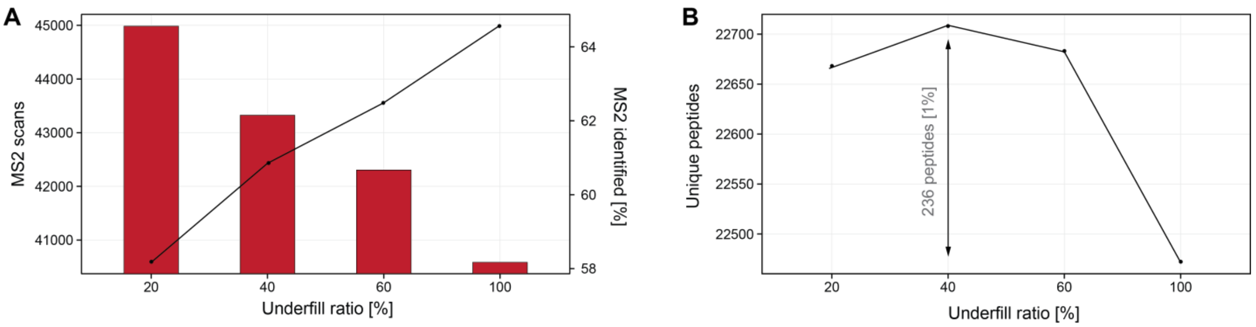

Underfil ratio titration series. Data collected with the standard settings, while varying the underfil ratio, on a HeLa whole cell lysate (see Supplementary Table 2 for further description). The chosen value for the underfil ratio affects the ion threshold for selecting an isotope for fragmentation. Data for underfil ratio 80 was excluded due to inconsistent behavior. **A.** The higher underfil ratios cause less MS2 scans to be recorded, however the identification rate increases. **B.** We find that the best sequencing success is at an underfil ratio of 40. Given the low differences as a result of differing underfil ratios, the method was set up with an underil ratio of 20 to maximize the number of MS2 scans being performed. The low difference between the best and worst setting can be explained from the high complexity of the sample, providing many highly abundant candidates at any given time during the gradient.

Supplementary Figure 6

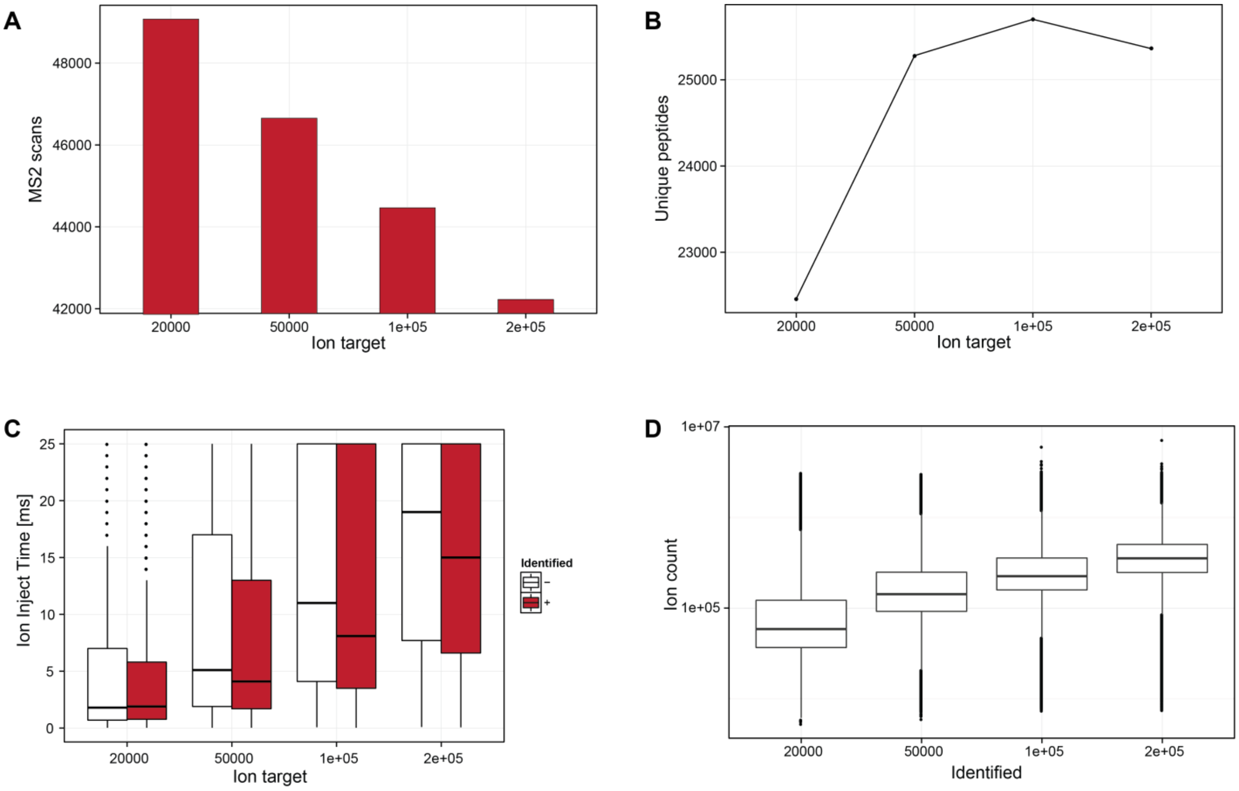

MS2 ion target titration series. Data collected with the standard settings, while varying the MS2 ion target value, on a HeLa whole cell lysate (see Supplementary Table 2 for further description). The chosen value for the target value affects the ion threshold for selecting an isotope for fragmentation. **A.** The number of fragmentation scans increases for the lower ion target values, as more isotope patterns will pass the ion threshold. **B.** The most optimal in terms of unique peptide sequences is however found at a target value of 1e5. **C.** With the higher target values the ions are required to be loaded for a longer time. **D.** With the higher target the number of ions used for fragmenting also increases.

Supplementary Figure 7

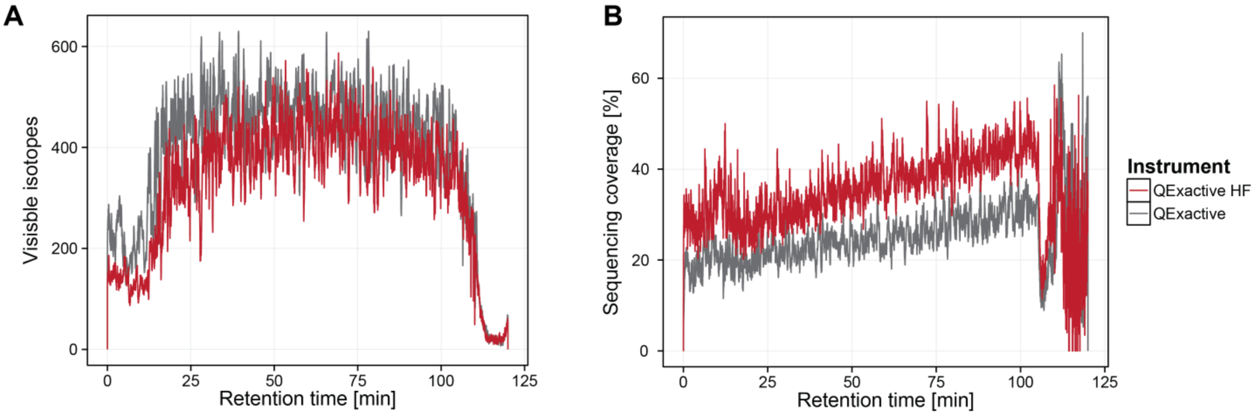

Peak-depth calculations. The peak-depth is calculated by first calculating the number of visible isotopes for each cycle. After sorting this list by descending intensity, the position of the isotope associated to each of the MS2 scans in the cycle is determined. **A.** The number of visible isotopes with charge state 2 and up at any given point in the gradient fluctuates between 400 and 500. This value is slightly elevated for the Q Exactive with the normal Orbitrap analyzer. A likely explanation for this is the marginally reduced resolution for the Orbitrap ultra-high field analyzer running in high speed mode (from 70k to 60k), resulting in a slight decrease of detectable isotope patterns. **B.** The high speed of the Orbitrap ultra-high field analyzer ensures that a higher percentage of available isotopes are sequenced.

Supplementary Figure 8

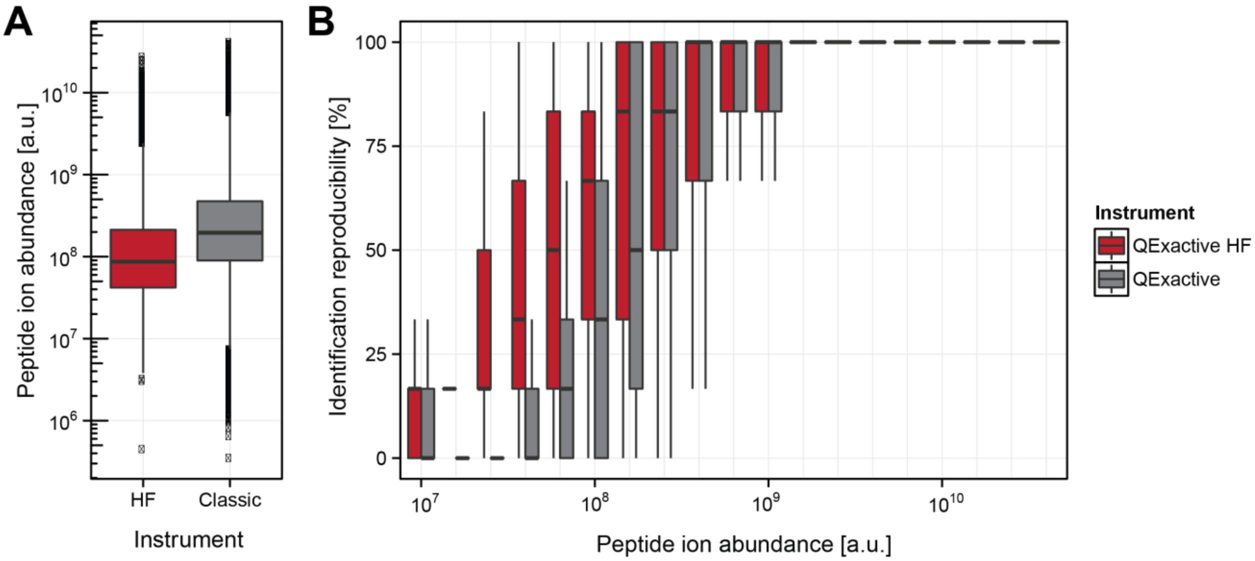

Dynamic range and reproducibility verification. A. Both instruments reliably sequence peptides over three orders of magnitude (excluding the outliers). The apparent drop in the peptide ion abundance can be attributed to different scaling of the reported intensity values, which the MaxQuant software can normalize at the protein level with the implemented MaxLFQ algorithm. **B.** To investigate whether the superior sequencing speed of the Q Exactive HF also benefits the identification reproducibility of the accessed set of peptides over 5 replicates we express the number of detections as a function of the peptide ion abundance. For both instruments we observe perfect identification reproducibility down to an ion abundance of 10e9. Below this value for both instruments the reproducibility starts to degrade. However, for the Q Exactive HF the percentage drops away less sharply, which can be attributed to the higher sequencing speed increasing the likelihood of the instrument accessing the same set of peptides at the formerly low end of the abundance scale.

Supplementary Figure 9

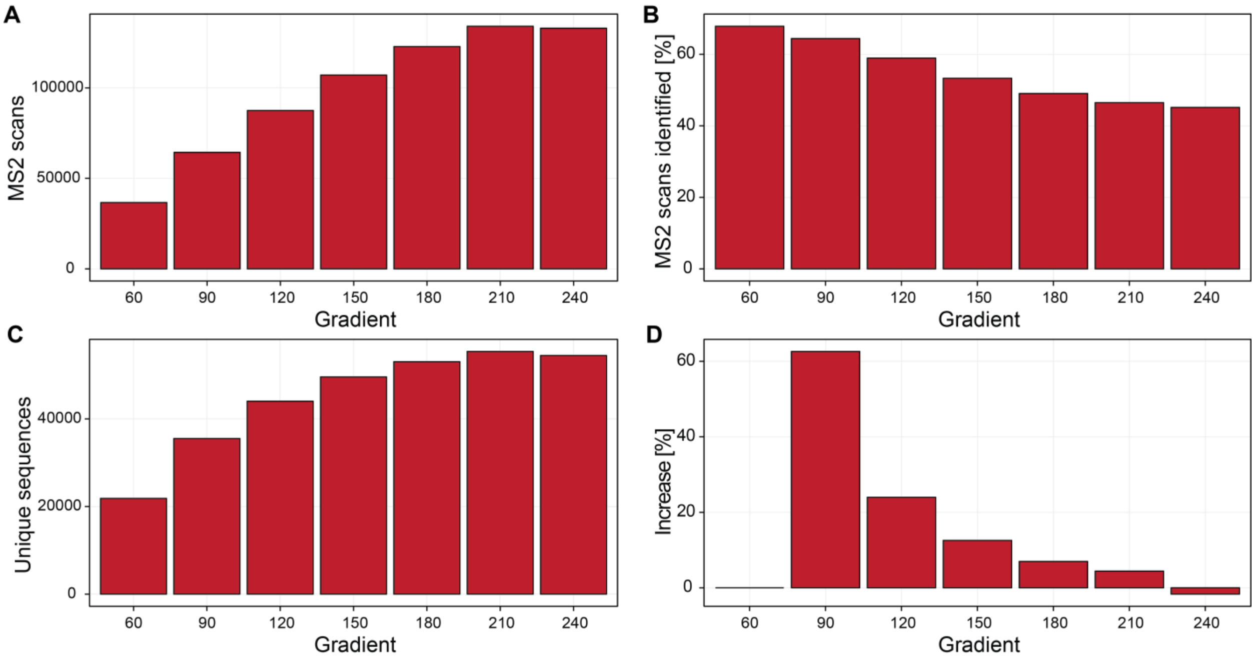

Gradient length titration series. Data was collected with the standard settings, with the only adaptation to the dynamic exclusion time to reflect the increase in peak-width for the longer gradients, on a HeLa whole cell lysate (see Supplementary Table 2 for further description). The range was chosen to encompass the most commonly used gradient lengths. **A.** With the longer gradient length we observe an almost linear increase in the number of performed MS2 scans, except for the 240 minutes which appears to finally have run out of sequencable candidates compared to the 210 minutes. **B.** The identification success-rate however decreases from almost 70% to well below 50%. **C.** This decrease results in a logarithmic decline in the extra number of unique peptides. **D.** By moving from 60 to 90 minutes an increase of 62% is achieved, which drops away to 23% by moving from 90 to 120 minutes and down to 12% when moving from 120 to 150 minutes. Based on these results we conclude that this particular sample a gradient length of 150 minutes is optimal, as longer gradients contribute less than 10% extra unique peptides. An increase that can also be achieved by measuring extra biological replicates from which, due to random sampling difference, the same increase can be obtained (those extra identifications can then be distributed between the measurements by the MaxQuant option Match-Between-Runs). A clear advantage of extra replicates is gained in improved statistical power during down-stream data analysis.

Supplementary Figure 10

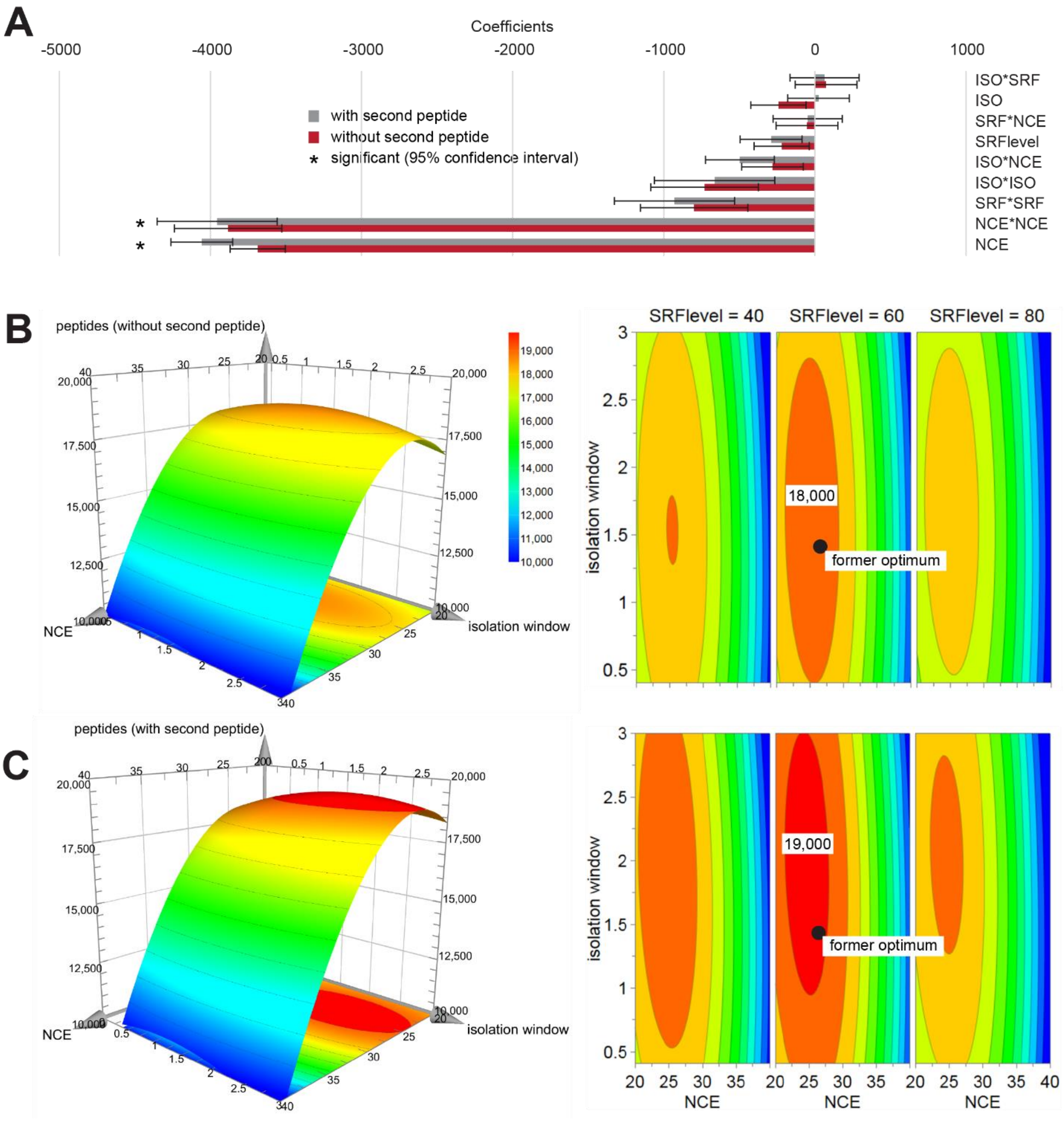

Parameter optimization verification. To verify whether the individually optimized parameters were chosen correctly and to detect whether any of those parameters are interacting (leading potentially to improved results) a Design of Experiment (DoE) was utilized (29). The analysis was performed with the software MODDE (Umetrics) optimizing the response of a sub-selection of user definable parameters (NCE, S-Lens RF level and isolation window size) on the mass spectrometric performance using the number of unique peptides as read out. The software provides for an optimization experiment (objective: Optimization (RSM)) a design of experiment (recommended design: CCF (star distance = 1)). Based on

the suggested design (which is sparse in the sense that not every combination is required to be measured) we ran 17 one hour gradients with the standard HeLa described in the manuscript. We utilized a combination of Quadratic Modeling and Multiple Linear Regression to uncover and quantify any pair-wise interactions. The output of the analysis is expressed as coefficients, where error-bars (standard error) smaller than the coefficient is better and can be visualized in a surface plot making it human readable. For the coefficients, a single term shows the linear and quadratic effect (e.g. iso and iso*iso, respectively) which that parameter has on the performance; two separate terms multiplied is the pairwise interaction between those terms. Model fitting was performed on the data processed with and without the MaxQuant option 'second peptide', which attempts to identify a second isotope pattern lying in the isolation window for already identified MS2 scans. **A.** We found that by far the largest effect (and the only statistically significant one as indicated by the '*') is induced by the NCE value, which controls the energy applied during fragmentation. When this energy is too high, the peptide is fragmented into many smaller, internal fragments which cannot be meaningfully interpreted. Although the other factors are not significant within the 95% confidence interval, the trend of the coefficients is as expected. The next two largest effects are induced by S-Lens RF-level and isolation window size (both quadratic effects). We find a single pair-wise interaction for isolation window and NCE, which seems to be stronger for the 'second peptide' results. This is as expected because a wider isolation window will lead to more second peptides in the fragmentation spectrum. Co-fragmentation of peptides could require a different energy to result in the most information rich fragmentation spectrum for all peptides involved. **B.** Surface plot visualizations of the results when processing the data without 'second peptide'. On the right-hand side the 4D surface plot is displayed, from which it can be read out that the S-Lens RF-level of 60 supplies the best performance. The data imply that for the interacting parameters NCE and isolation window an inverse relationship holds (i.e. larger isolation windows require lower fragmentation energy). To verify whether this effect is significant the parameter range for the NCE would need to be decreased. The black dot in this 4D surface plot indicates the previously individually optimized values for the chosen parameters (NCE=27, S-Lens=60, isolation window=1.4), which falls inside the optimal area. The 3D surface plot on the left-hand side for the S-Lens RF-level of 60 is provided as a reading guide for the 4D surface plot. **C.** Surface plot visualizations of the results when processing the data with the MaxQuant option 'second peptide'. While the overall impact of the parameters is similar, the performance appears to benefit from a slightly wider isolation window.

Based on the above results we conclude that the previously found values (NCE=27, S-Lens=60, isolation window=1.4) are optimal, as they fall within the optimal area for both with as well as without 'second peptide' indicated by the design of experiment evaluation. As there is a clear fit to the previously independently optimized values and we detected only a rather weak set of interacting parameters we conclude that significantly increased performance cannot be achieved by varying this sub-set of parameters. However, we note that due to its ability to handle sparse data, the DOE approach can be highly efficient for experimental parameter optimization for different sample and chromatography types, given that large ranges can be investigated without accumulating many mass spectrometry measurements.

Supplementary Figure 11

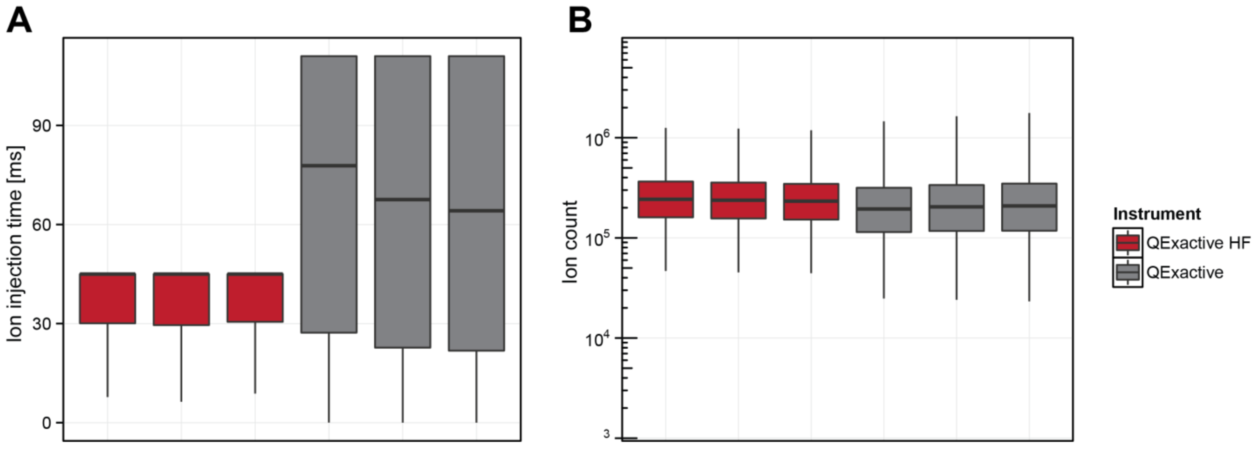

Achieved AGC target comparison for the phosphorylation comparison. A. The maximum ion injection time was restricted to 45 ms for the HF and 111 ms for the QExactive. For the QExactive HF this means that most of the scans have an injection time between 30 and 45 ms with a median of 45 ms (i.e. ‘maxing out’). For the QExactive we find a range of times between 30 and 111 ms, with a median of 70 ms. **B.** Even with the restricted ion injection times both instruments achieve the set AGC target value of 1e5 ions (or even a little above). The HF is doing this in a shorter time due to the improved quadrupole transmission at small isolation windows, allowing for a higher scan speed and consequently more identifications.
