# Supplementary figures and images for: The Q Exactive HF, a Benchtop Mass Spectrometer with a Pre-filter, High-performance Quadrupole and an Ultra-high-field Orbitrap Analyzer
Source: Mol Cell Proteomics. 2014 Oct 30;13(12):3698–708. doi: 10.1074/mcp.M114.043489 (PMC4256516; doi:10.1074/mcp.M114.043489)

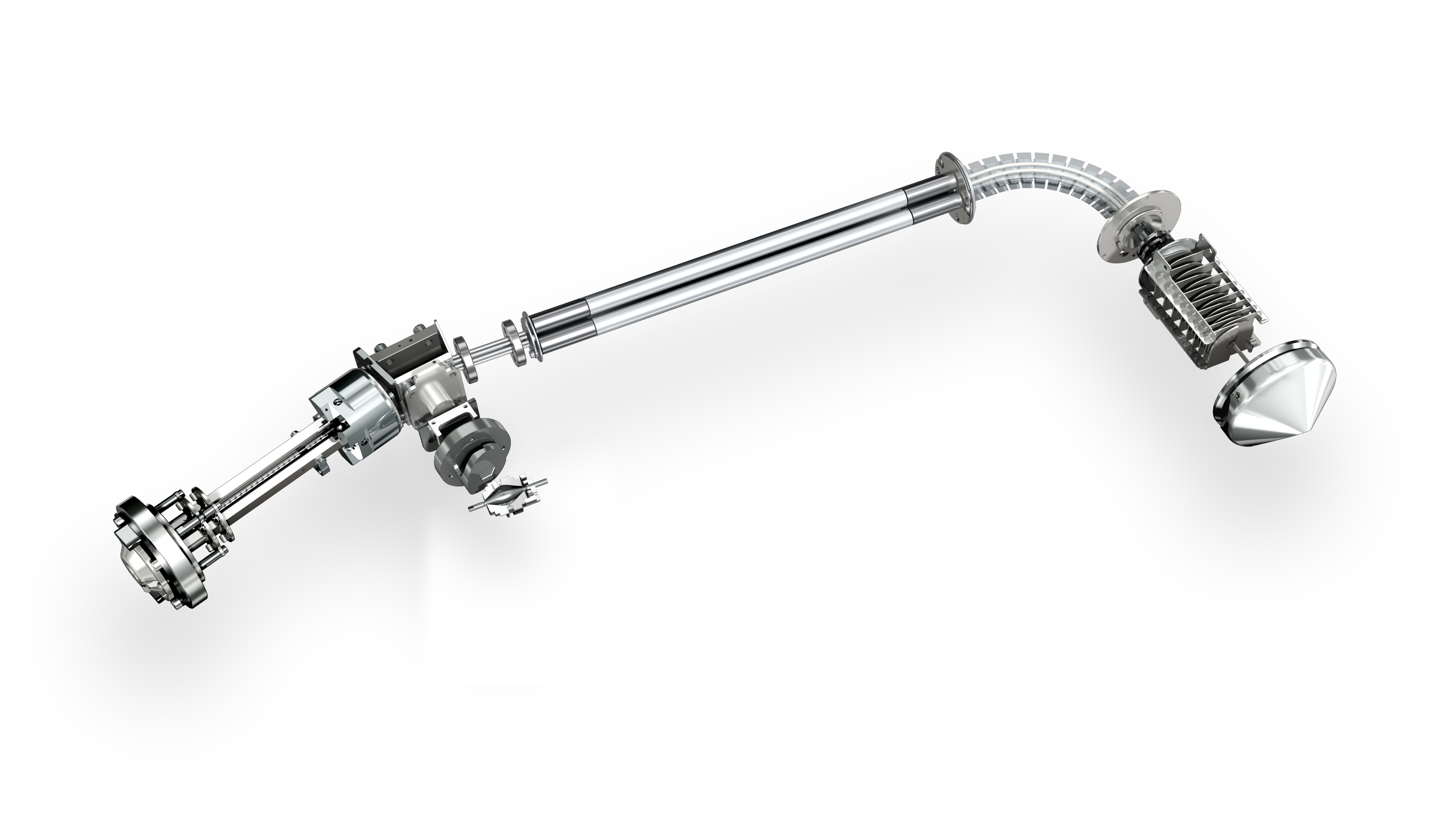

Supplement: Supplemental Data [file supp_M114.043489_mcp.M114.043489-3.zip › QexactiveHF internal_May6_2.png]
